# Human papillomavirus-related syntaxin 11 reprograms tumor-associated macrophages to induce breast cancer cell apoptosis via PI3K/AKT signaling

**DOI:** 10.1186/s10020-025-01325-z

**Published:** 2025-09-02

**Authors:** Chuan Hu, Tingting Hu, Jian Wen, Zengrong Jia

**Affiliations:** 1 Department of Interventional Therapy, Zhejiang Cancer Hospital, Institute of Basic Medicine and Cancer (IBMC), Chinese Academy of Sciences, Hangzhou, 310022 China; 2 School of Medicine, Zhejiang University, Hangzhou, 310009 China; 3 Department of Cardiology of The Second Affiliated Hospital, School of Medicine, Zhejiang University, Hangzhou, 310009 China; 4 State Key Laboratory of Transvascular Implantation Devices, Zhejiang University, Hangzhou, 310009 China; 5 Heart Regeneration and repair Key Laboratiry of Zhejiang Province, Hangzhou, 310009 China; 6 Department of Oncology, The First Affiliated Hospital of Wenzhou Medical University, Wenzhou, 325000 China; 7 Wenzhou Medical University, Wenzhou, 325000 China; 8 Department of Thyroid, The First Affiliated Hospital of Wenzhou Medical University, Wenzhou, 325000 China

## Abstract

**Background:**

Human papillomavirus (HPV) is closely associated with tumor progression and the tumor microenvironment (TME), but its role in breast cancer (BC), which can be affected by HPV, has not been reported.

**Methods:**

Ten independent BC cohorts were included to generate two HPV-related gene-based signatures. The CIBERSORT and ESTIMATE algorithms were used to quantify the immune cell fraction and TME scores, and the correlations between HPV-related gene-based signatures and scores were analyzed. The expression patterns and clinical significance of STX11 were determined through bioinformatics analysis, and its effects on modulating tumor-associated macrophages (TAMs) were confirmed by real-time qPCR and Western blotting. The anticancer role of STX11 in macrophages and its underlying mechanisms were analyzed in vitro and in vivo.

**Results:**

Two novel HPV-related gene-based signatures were established that can effectively predict the overall survival and disease-free survival of patients with BC. HPV-related gene-based signatures were significantly associated with the immune score and 19 types of immune cells in BC tissues. STX11 was downregulated in BC and was associated with favorable clinical prognosis, and it was expressed mainly in M1 TAMs. Mechanistically, STX11 promoted the M1 polarization of macrophages, and macrophages overexpressing STX11 can inhibit BC proliferation and migration by regulating the PI3K–AKT pathway. In orthotopic BC models, macrophages overexpressing STX11 significantly suppressed tumor growth.

**Conclusions:**

HPV-related risk signatures were constructed, which showed prognostic predictive ability for patients with BC. STX11 is associated with a favorable prognosis in patients with BC and facilitates M1 polarization, and macrophages overexpressing STX11 can inhibit BC malignancy by regulating the PI3K–AKT pathway, suggesting its role as a potential immunotherapeutic candidate.

**Supplementary Information:**

The online version contains supplementary material available at 10.1186/s10020-025-01325-z.

## Introduction

Breast cancer (BC) is the most common malignant tumor in female patients, with more than 2.3 million new cases and 685,000 deaths from BC in the latest report (Arnold et al. 2022). Unfortunately, it is estimated that the burden of BC will increase to more than three million new cases and one million deaths by 2040 because of population growth and aging alone (Arnold et al. 2022). Currently, the treatments for BC include surgery, radiotherapy, chemotherapy, endocrine therapy and targeted therapy (Haddad et al. 2024). However, the prognosis of patients with BC is still unfavorable, and the outcomes vary significantly among patients. Thus, it is necessary to identify new therapeutic targets and develop prognostic markers for patients with BC.

Human papilloma virus (HPV) easily infects the human epidermis and mucous squamous epithelium and plays an important role in the pathogenesis and progression of tumors (Araldi et al. 2018; Szymonowicz and Chen 2020). Currently, studies on the relationship between HPV and tumors have focused mainly on cervical cancer and head and neck tumors (Malagón et al. 2024; Falcaro et al. 2024). However, oncologists have reported a potential relationship between HPV and BC (Heng et al. 2009). In 2012, Priscyla et al. (Priscyla Waleska et al. 2012) performed a systematic review including 29 primary studies and 2211 samples. The prevalence of HPV in patients with BC was 23.0% (95% CI: 21.2–24.8%)^8^. Moreover, HPV infection has been confirmed to be related to clinical parameters and affects the prognosis of patients with BC (Bae and Kim 2016; Liu et al. 2018). Therefore, HPV certainly plays a role in BC, but the mechanism is still unclear. Recently, sequencing technology has been widely used to reveal the carcinogenesis and progression of tumors. However, until now, no studies have reported the relationship between HPV and the gene expression patterns of BC.

Tumor-associated macrophages (TAMs) are among the main immune cells in the tumor microenvironment (TME) and are divided into M1 (proinflammatory) and M2 (anti-inflammatory) macrophages (Pittet et al. 2022; Maller et al. 2021). In tumor tissues, tumor cells secrete a variety of substances to hijack TAMs into the M2 type, thereby protecting tumor tissue from the killing of immune cells (He et al. 2023; Lu et al. 2022; Deng et al. 2024). Therefore, reprogramming M2 macrophages to M1 macrophages is a potential cancer treatment (Zhang et al. 2023; Wang et al. 2024). In recent years, the intratumor microbiome, including bacteria, viruses and fungi, has not been shown to be inert but has specific roles, including regulating the infiltration and polarization of macrophages (Ma et al. 2024; Sun et al. 2023; Liu et al. 2023). Ma et al. (Ma et al. 2024) reported that intratumor microbiome-derived butyrate promotes lung cancer metastasis by inducing M2 macrophage polarization, which indicates that the intratumor microbiome may regulate the polarization of macrophages and influence tumor fate. Although there is currently no evidence that HPV in BC directly regulates TAM polarization, researchers have reported that HPV-positive HNSCC-derived exosomal miR-9 induces macrophage M1 polarization and increases tumor radiosensitivity (Tong et al. 2020). Therefore, further study of the role of HPV in the regulation of the TME may facilitate the development of novel treatments for patients with BC.

In this study, we used a series of gene expression profiles and the OncoDB database to comprehensively analyze the role of HPV-related genes in patients with BC (Tang et al. 2021). We revealed that HPV-related gene-based signatures are robust prognostic biomarkers for patients with BC and are significantly associated with the tumor microenvironment of BC tissues. Moreover, we demonstrated that STX11 promoted the polarization of antitumor M1-like macrophages in BC and that macrophages overexpressing STX11 inhibited tumor progression by regulating the PI3K–AKT signaling pathway.

## Materials and methods

### Download and processing of data from patients with BC for bioinformatics analyses

The RNA sequencing data of the BC patient (TCGA-BRCA) cohort were downloaded from UCSC Xena (https://xenabrowser.net/datapages/), and the corresponding clinical information was obtained from cBioPortal (https://www.cbioportal.org). The microarray data and clinical information, including datasets GSE42568, GSE70947, GSE1456, GSE20685, GSE58812, GSE31448, GSE45255, GSE2034 and GSE12276, were downloaded from the Gene Expression Omnibus (GEO, https://www.ncbi.nlm.nih.gov/geo/). A total of 1143 HPV-related genes were obtained from the OncoDB database, an interactive online database for the analysis of gene expression and viral infection in cancer (Tang et al. 2021). The TCGA-BRCA cohort was used for developing prognostic signatures; GSE1456, GSE20685 and GSE58812 were merged as a combined cohort for validating the overall survival (OS) signature; and GSE31448 and GSE45255 were merged as a combined cohort for validating the disease-free survival (DFS) signature. In the process of merging cohorts, “sva” packages were used to remove batch effects between datasets.

### Development and validation of the HPV-based signature for patients with BC

The differential analyses were performed independently in three cohorts containing adjacent normal tissues, including TCGA-BRCA, GSE70947 and GSE42568. Genes with|logFC|≥1 and FDR < 0.05 were confirmed as differentially expressed genes (DEGs), and the intersecting genes among the three cohorts were defined as cancer- and HPV-related genes (CAHGs) and selected for further analyses. Protein‒protein interaction (PPI) analysis was performed by the STRING database (Szklarczyk et al. 2014). The enrichment analyses of CAHGs, including Gene Ontology (GO) and pathway (Kyoto Encyclopedia of Genes and Genomes (KEGG) and Hallmark), were performed in Metascape, an innovative web-based portal designed to provide comprehensive gene list annotations and resources for experimental biologists (Zhou et al. 2019).

Two HPV signatures, OS and DFS, were established based on the CAHGs with univariate and multivariate Cox analyses. The riskScores of all patients were calculated using the following equation:

$$\begin{aligned}\:riskScore=&Coefficient1*expression1\:+\:...\\&+\:CoefficientN*expressionN \end{aligned}$$

All patients were divided into low- or high-risk groups according to the median riskScore, and Kaplan‒Meier (K‒M) survival curves were established to evaluate survival outcomes. Time-dependent receiver operating characteristic (ROC) curves were generated, and area under the curve (AUC) values at five and seven years were plotted. In the validation cohort, the riskScores were calculated using the equation established in the training cohort. The median riskScores were subsequently identified, and all patients in the validation cohort were divided into two risk groups. Survival and ROC curves were generated to confirm that the HPV signatures had high precision and fit. To elucidate the independent performance of the HPV-based signatures in predicting the prognosis of patients with BC, univariate Cox analysis was performed, and clinical parameters with *P* < 0.05 were further incorporated into multivariate Cox analysis. Finally, two nomograms incorporating independent prognostic biomarkers were established.

Owing to the potential correlation between HPV and the TME in tumors, we investigated the associations between the HPV-based signature and TME features, including TME scores, immune cell infiltration, and immune checkpoints. TME scores were calculated with the Estimation of STromal and Immune cells in MAlignant Tumours using Expression data (ESTIMATE) algorithm (Yoshihara et al. 2013). The immune cell infiltration data were analyzed using the single-sample gene set enrichment analysis (ssGSEA) algorithm based on the RNA sequencing data. For immune checkpoints, PDCD1, CD274, LAG3, CTLA4, HAVCR2, and TIGIT were analyzed. In this section, we also performed gene set variation analysis (GSVA) to explore mechanism differences between low- and high-risk groups (Hänzelmann et al. 2013). The gene sets enrolled in the GSVA analyses included “h.all.v2022.1.Hs.symbols” and “c2.cp.kegg.v2022.1.Hs.symbols”.

### Bioinformatics analyses of STX11 with bulk and single-cell sequencing data

In the present study, two CAHGs, MMP1 and STX11, were simultaneously included in both the OS and the DFS signatures. Because the association between MMP1 and BC has been widely reported, we selected STX11, a gene that has been less studied, for further study (Ma et al. 2017; Farooqui et al. 2015; Mannello 2011; McGowan and Duffy 2008; Argote Camacho et al. 2021; Kim et al. 2022). The expression of STX11 according to different clinical characteristics was compared. Survival outcomes, including OS, DFS, metastasis-free survival (MFS), and bone metastasis-free survival (BMFS), were compared between the low- and high-STX11 expression groups, and K‒M survival curves were generated for patients with BC. Moreover, the associations between the expression of STX11 and the TME score, immune cell infiltration, immune checkpoints, and enrichment analyses were analyzed. In addition to bulk sequencing data, we performed single-cell sequencing analyses with the TISCH2 and Tumor Immunity General Resource databases (Han et al. 2022; Chen et al. 2022).

A literature search revealed that STX11 is a novel biomarker, and its relationship with solid tumors has rarely been reported. Therefore, we performed pancancer analyses to systematically elucidate the role of STX11 in solid tumors. The gene expression profiles and corresponding follow-up data of 30 solid tumors were obtained from UCSC Xena. The expression patterns, survival relationships, TME relationships, and correlations with the genomic heterogeneity of STX11 were analyzed.

### Cell culture and transfection

Human BC cell lines and normal mammary epithelial cells, including MDA-MB-231, MCF-7 and THP-1 cells, were purchased from Fuheng Biology (Shanghai, China). MDA-MB-231 and MCF-7 cells were cultured in DMEM (Gibco) supplemented with 10% FBS (CellMax) and 1% penicillin‒streptomycin. THP-1 cells were cultured in RPMI-1640 (Gibco) supplemented with 10% FBS (CellMax) and 1% penicillin‒streptomycin. All the cell lines were cultured at 37 °C in a humidified incubator containing 5% CO_2_.

The full-length sequence of STX11 was subcloned and inserted into a pcDNA3.1 (+) vector. All the plasmids were validated by DNA sequencing. Small-interfering RNA (siRNA) oligonucleotides against STX11 were purchased from RiboBio (Guangzhou, China). The sequence of the siRNA used was 5’-CATCGAGCTCAACGTACAA-3’ (si-STX11). Transfection was performed using the Lipofectamine 2000 reagent according to the manufacturer’s instructions.

### CCK-8

The viability of the tumor cells was analyzed using a Cell Counting Kit-8 (CCK8, APExBIO, Houston, USA). Briefly, a total of 5000 MCF-7 or MDA-MB-231 cells/well were cultured in 96-well plates for 1, 3, 5 or 7 days. The cells in each well were treated with 10 µL/well CCK-8 reagent, and their viability was determined by measuring the absorbance of individual wells at 450 nm using a microplate reader. CCK-8 assays were performed in triplicate using three independent cell cultures (*n* = 3 biological replicates), with each time point measured in technical triplicate.

### Colon formation

For the colony formation assays, the MCF-7 and MDA-MB-231 cells were digested, resuspended and counted. The cells were stored in 12-well plates at a density of 500 or 300 cells/well. The culture time of the cells was 14 days. Then, the cells were fixed with 4% paraformaldehyde for 30 min and stained with crystal violet for 30 min. Images of the colonies were obtained with a scanner, and the colonies were counted. Colony formation assays were performed in triplicate using three independent cell cultures (*n* = 3 biological replicates).

### Transwell migration assays

Transwell assays were performed in 24-well Transwell plates (8 μm. Corning, USA). Briefly, MCF-7 and MDA-MB-231 cells were cultured in FBS-free medium in the top chamber, and complete medium was added to the bottom chambers for 24 h. The cells on the upper surface of the top chamber were removed with a cotton ball. The cells that had invaded the lower surface of the top chamber were fixed with 4% paraformaldehyde and stained with crystal violet, and were then imaged under a light microscope. The migrated cells were enumerated in three fields of view/membrane. Transwell migration assays were repeated three times (*n* = 3 biological replicates).

### Real-time qPCR

The total RNA was extracted from the cells using TRIzol (Agbio, Changsha, CHN), and cDNA was synthesized using a reverse transcription reagent kit (Agbio, Changsha, CHN) according to the manufacturer’s recommendations. Information about the primers used is shown in Table S1. The RT‒PCR analysis included three biological replicates.

### Western blotting

Cells were lysed in radioimmunoprecipitation assay (RIPA) buffer and centrifuged (12,000 × g at 4 °C for 20 min). The protein concentrations were subsequently determined using a BCA kit, and the lysates were separated by sodium dodecyl SDS‒PAGE on 10% gels and transferred to PVDF membranes. The membranes were blocked with 5% BSA in TBST and incubated with primary antibodies (anti-GAPDH (Proteintech, 60004-1-Ig), anti-STX11 (Proteintech, #13301-1-AP), anti-PI3K (ABclonal, # A23303PM), anti-AKT (Proteintech, #60203-2-Ig) and anti-p-AKT (Proteintech, #80455-1-RR)) at 4 °C overnight. The membranes were washed with Tris-buffered saline containing 0.05% Tween-20, incubated with peroxidase-conjugated anti-rabbit IgG or anti-mouse secondary antibodies (Fude Biological Technology, Hangzhou, China) and washed once more. The blots were developed using an ECL substrate solution (Share-bio, Shang Hai). Western blots were performed with three independent lysates (*n* = 3 biological replicates), and representative images are shown.

### Flow cytometry

Cancer cells were harvested from 6-well plates after coculture with THP-1 cells for 48 h and washed twice with PBS, single-cell suspensions were generated, and then, the cells were incubated with FITC and 7-AAD (Yeasen, #40310ES) for 10 min at room temperature. The stained cells were then resuspended in 400 µL of flow buffer on a flow cytometer (Beckman, USA), and FlowJo software (FlowJo, USA) was used for data analysis. The gating strategy for flow cytometry is shown in Figure S1.

### In vivo analyses

Female nude mice (6 weeks old, 18–20 g) were selected for tumor-bearing experiments in our study. All the experimental procedures were performed in accordance with the protocols and ethical regulations approved by the Institutional Animal Care and Use Committee, Zhejiang Center of Laboratory Animals (ZJCLA-IACUC-20010503). Prior to xenografting, the mice were randomly divided into three groups (*n* = 5/group). MCF-7-*luc* cells (5 × 10^6^ per mouse) were mixed with THP-1-derived Mφ macrophages (1 × 10^6^ per mouse) in Matrigel solution and injected into the fourth left mammary fat pad. Tumor growth was observed daily, and photographs were taken with an in vivo imaging system. The tumor volumes in mice were calculated (V = 0.5×length×width^2^) every 5 days. After 15 days, the tumor tissues were collected and fixed with 4% paraformaldehyde for further use.

### Immunohistochemistry (IHC)

First, the tissue sections were deparaffinized and then rehydrated with graded ethanol. For antigen repair, the sections were microwaved for 15 min in EDTA, immersed in 3% hydrogen peroxide to quench endogenous peroxidase, and blocked with goat serum at RT for 1 h. Next, the sections were probed with primary antibody against TUNEL at 4 °C overnight and then labeled with an HRP-conjugated secondary antibody at room temperature for 1 h. Finally, immunostaining was visualized with diaminobenzidine, and the slices were counterstained with hematoxylin.

### Statistical analysis

All the statistical analyses were conducted using R software (v.4.1.3) or GraphPad Prism (v.10.1.2). Student’s t test or the Wilcoxon rank-sum test was used to compare data between two groups, and one-way ANOVA was used to compare data. Correlation analysis was performed using Spearman tests. Univariate and multivariate Cox regression models were used to examine the independent prognostic factors. The log-rank test was performed to compare the survival curves between groups. A two-tailed p value < 0.05 was considered statistically significant for all tests.

## Results

### Overview of HPV-related genes in patients with BC

**Fig. 1:**
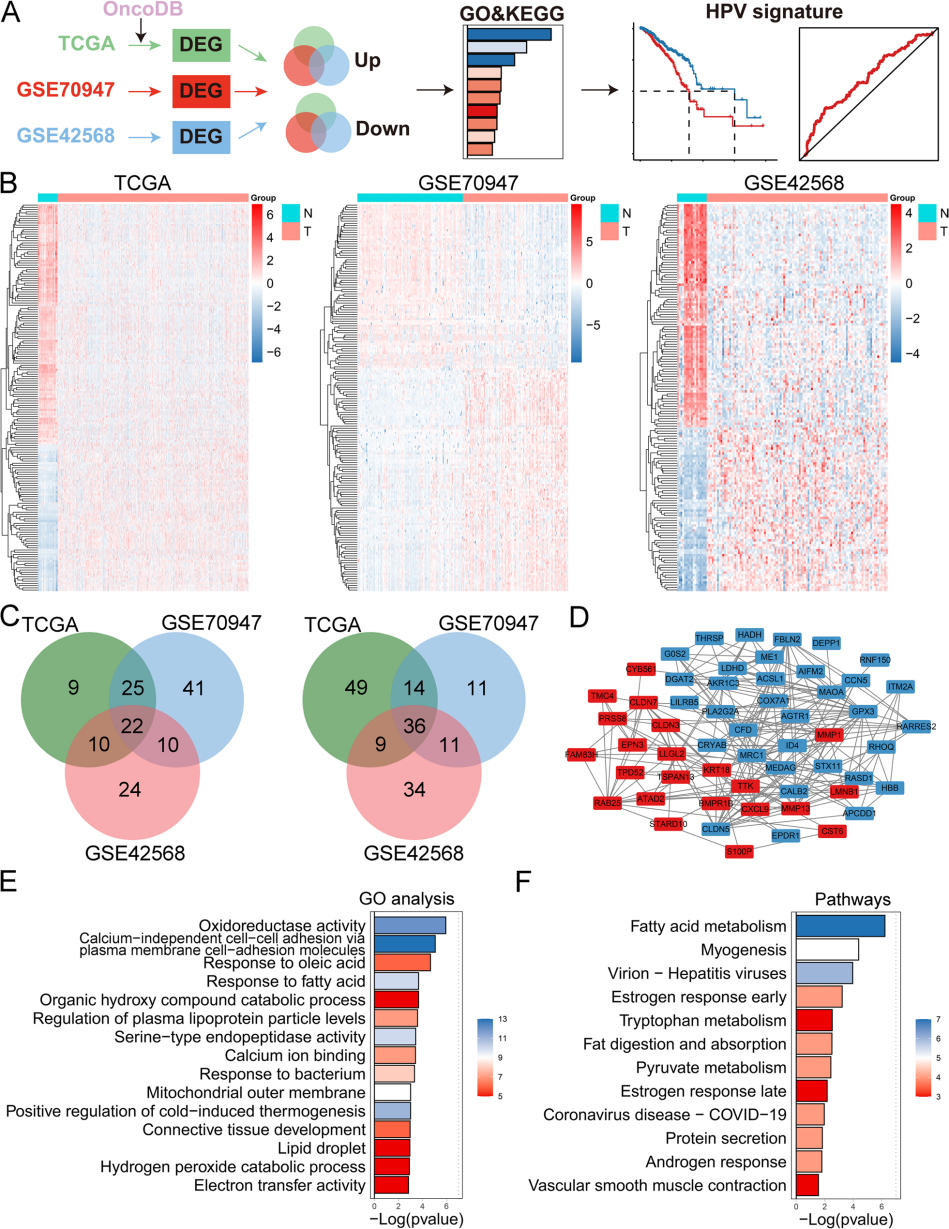
**A** Flow chart of HPV signature establishment. **B** Heatmaps showing the expression levels of differentially expressed genes in the TCGA, GSE70947 and GSE42568 cohorts. **C** Venn diagram showing intersectional cancer-related HPV-related genes among the TCGA, GSE70947 and GSE42568 cohorts. **F** Protein‒protein interaction network of cancer-related HPV-related genes. **G** Gene ontology analysis of cancer-related HPV-related genes. **H** Pathway analysis of cancer-related HPV-related genes

The schematic for the partial bioinformatics analyses is shown in Fig. 1A. A total of 174, 170 and 156 HPV-related genes were identified as DEGs in the TCGA, GSE70947 and GSE42568 datasets, respectively (Fig. 1B). Among these genes, 22 CAHGs were intersecting genes whose expression levels were upregulated among the three cohorts, and 36 CAHGs were intersecting genes whose expression levels were downregulated (Fig. 1C). The PPI network of these CAHGs is shown in Fig. 1D. Functional analyses revealed that these genes were correlated with fatty acid metabolism and inflammatory-related pathways, among other processes (Fig. 1E and F). Only 58 genes were identified as DEGs, accounting for a lower proportion of all HPV-related genes, but these CAHGs were significantly associated with immune, metabolism and cellular functions, suggesting important roles for these genes in the progression of BC.

### HPV-related signatures are independent prognostic biomarkers for patients with BC

**Fig. 2:**
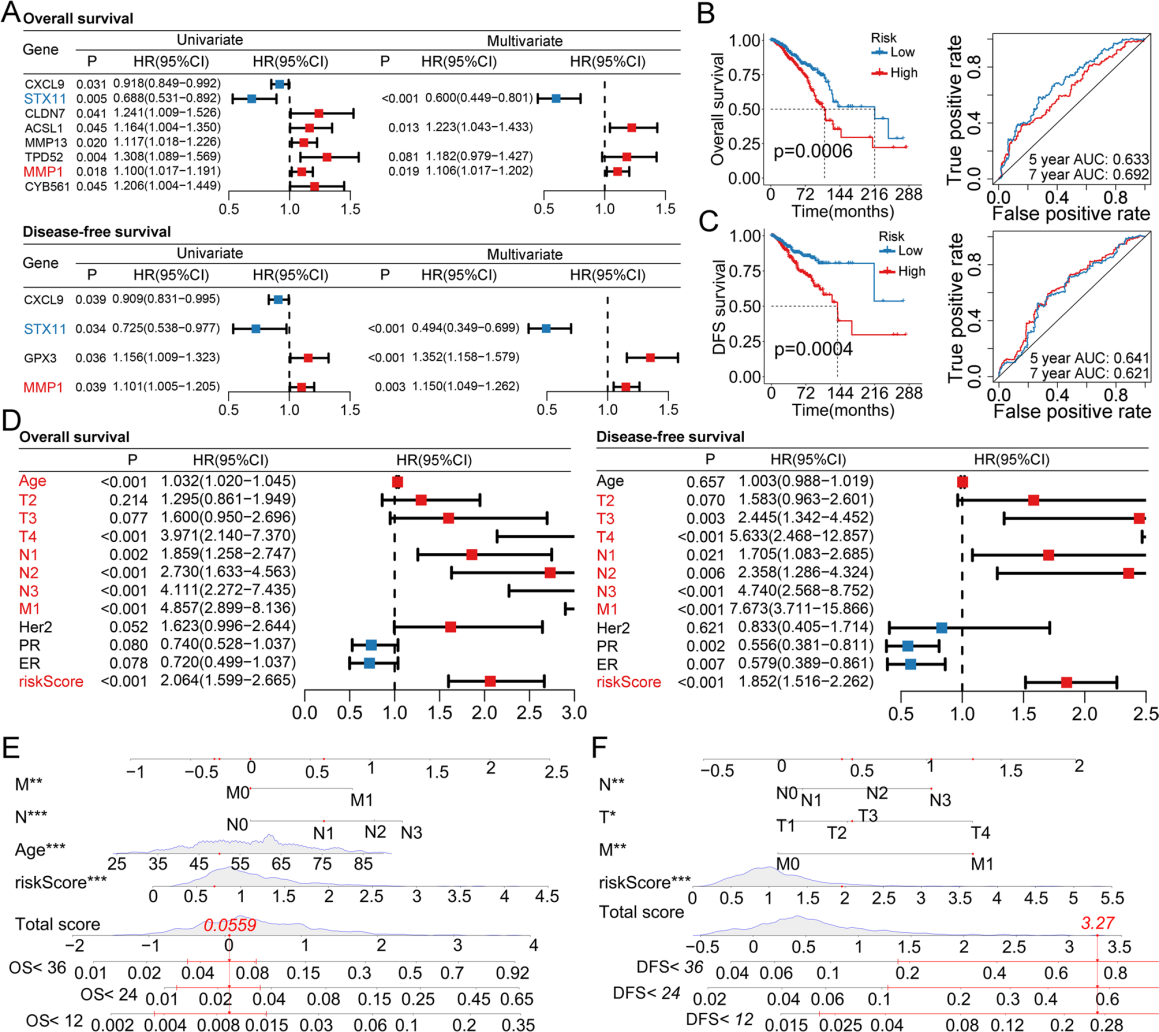
Establishment and validation of two HPV-related signatures for patients with BC. **A** Univariate and multivariate Cox analyses of OS and DFS for cancer-related HPV-related genes. **B** Survival and time-dependent ROC curves of the OS signature in the developing cohort. **C** Survival and time-dependent ROC curves of the DFS signature in the developing cohort. **D** Univariate analyses of OS and DFS for the HPV-related signature and clinical parameters. **E** Nomogram combining the HPV-related signature and prognostic clinical parameters for predicting the OS of patients with BC. **F** Nomogram combining the HPV-related signature and prognostic clinical parameters for predicting the DFS of patients with BC

Among the 58 CAHGs, eight and four genes were confirmed as OS- and DFS-related biomarkers, respectively, in patients with BC (Fig. 2A). Four and three CAHGs were subsequently selected as optimal prognostic biomarkers and were used for developing OS and DFS signatures, respectively (Fig. 2A). Survival analyses indicated that patients in the high-risk groups had poorer OS and DFS than those in the low-risk groups (Fig. 2B and 2C). Time-dependent ROC curves revealed that the AUC values for the OS signature were 0.633 and 0.692 at 5 and 7 years, respectively (Fig. 2B). The AUC values for the 5- and 7-year DFS signatures were 0.641 and 0.621, respectively (Fig. 2C). Subtype analyses revealed that HPV-related signatures were significantly associated with OS in the luminal and basal subtypes but were associated with DFS in the luminal and HER2 subtypes (Table S2). In the validation cohort, the results revealed that the HPV-related signatures successfully stratified patients into two risk groups (*p*<0.05) (Figures S2 and S3). Time-dependent ROC curves confirmed that both signatures still strongly differentiated in the validation cohort, and the AUC values of both signatures were greater than 0.600 (Figure S2 and S3). Overall, these results showed that HPV-related signatures are robust prognostic tools for patients with BC.

We further tested the independent performance of the HPV-related signatures. Univariate Cox analyses revealed that age, T stage, N stage, M stage and HPV-related signatures were OS-related clinical parameters, whereas age, T stage, N stage, M stage, PR status, ER status, and the riskScore based on the HPV-related signature were DFS-related clinical parameters (Fig. 2D). Further multivariate Cox analyses indicated that both signatures were independent prognostic biomarkers for patients with BC (Table S3 and S4). Moreover, age, N stage, and M stage were confirmed as independent OS-related biomarkers, and T stage, N stage, and M stage were confirmed as independent DFS-related biomarkers for patients with BC (Table S3 and S4). Finally, two nomograms were established by incorporating all the independent prognostic parameters (Fig. 2E and 2F).

###  HPV-related signatures are significantly associated with the TME, inflammation, and ICI therapy in patients with BC

**Fig. 3:**
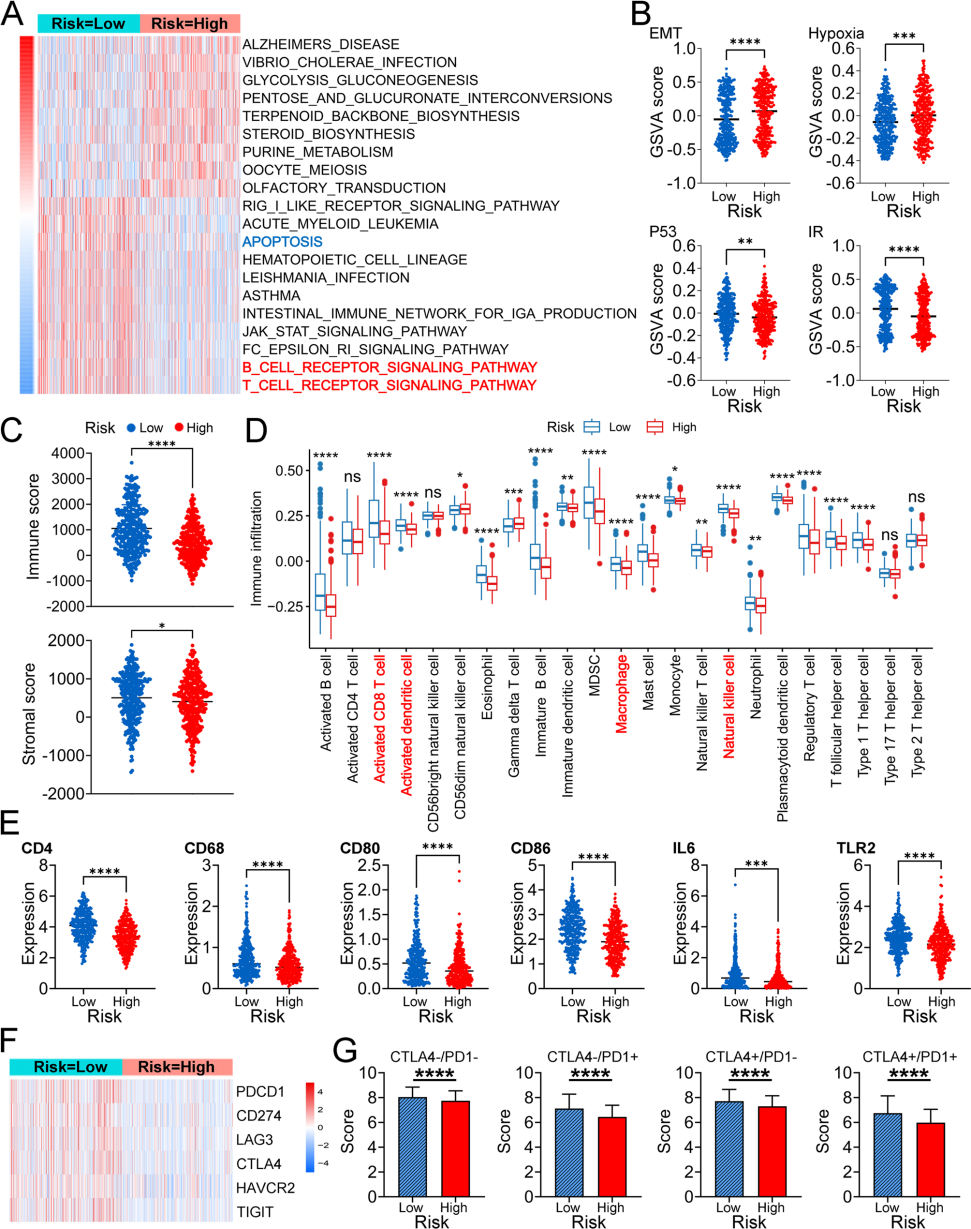
Comprehensive analyses of the correlations between HPV-related signatures and the tumor microenvironment, inflammation, and ICI therapy. **A** GSVA between the low- and high-risk groups. (**B**) GSVA of epithelial–mesenchymal transition, hypoxia, the P53 pathway and the inflammatory response between the low- and high-risk groups. **C** Immune and stromal scores between the low- and high-risk groups. **D** Immune cell infiltration between the low- and high-risk groups. **E** Expression profiles of five immunological genes between the low- and high-risk groups. **F** Expression profiles of five immune checkpoints between the low- and high-risk groups. **G** TCIA scores between the low- and high-risk groups; **** *P*<0.0001 ***P<0.001 ** *P*<0.01 *P<0.05

We employed an innovative approach to stratify patients into two groups. When a patient was classified as having a low risk for both OS and DFS, the patient was considered to have a good prognosis (Risk=Low), and when a patient was classified as having a high risk for both OS and DFS, the patient was considered to have a poor prognosis (Risk=High). We first analyzed the pathway differences between the two risk groups. Patients with BC in the high-risk group had lower apoptosis, B-cell receptor signaling pathway, and T-cell receptor signaling pathway scores than patients in the low-risk group did (Fig. 3A). In addition, patients with BC in the high-risk group presented increased epithelial‒mesenchymal transition (EMT) and hypoxia scores but decreased P53 pathway and inflammatory response scores (Fig. 3B). These results suggest that the HPV signature is not only related to prognosis but also affects the immune microenvironment of BC. Tumor microenvironment scores, including immune scores and stromal scores, were significantly different between the groups, indicating that the purity was greater in patients in the high-risk group (Fig. 3C). In terms of immune cells, the ssGSEA results revealed that high-risk patients had lower infiltration of immune cells, especially CD8^+^T cells, activated dendritic cells, natural killer T cells, and natural killer cells; these cells are important anticancer immune cells (Fig. 3D). In addition, the expression levels of several immunological genes and immune checkpoints were significantly lower in high-risk patients than in high-risk patients (Fig.3E and F). Finally, four scores downloaded from the TCIA (which can be used for predicting immunotherapy efficacy) were significantly different between the low- and high-risk groups (Fig. 3G). Overall, these results suggest that patients in the high-risk group are in an immunosuppressed state, which may be one of the factors contributing to the poor prognosis.

### STX11 is closely related to the prognosis, clinical characteristics and TME of patients with BC

**Fig. 4:**
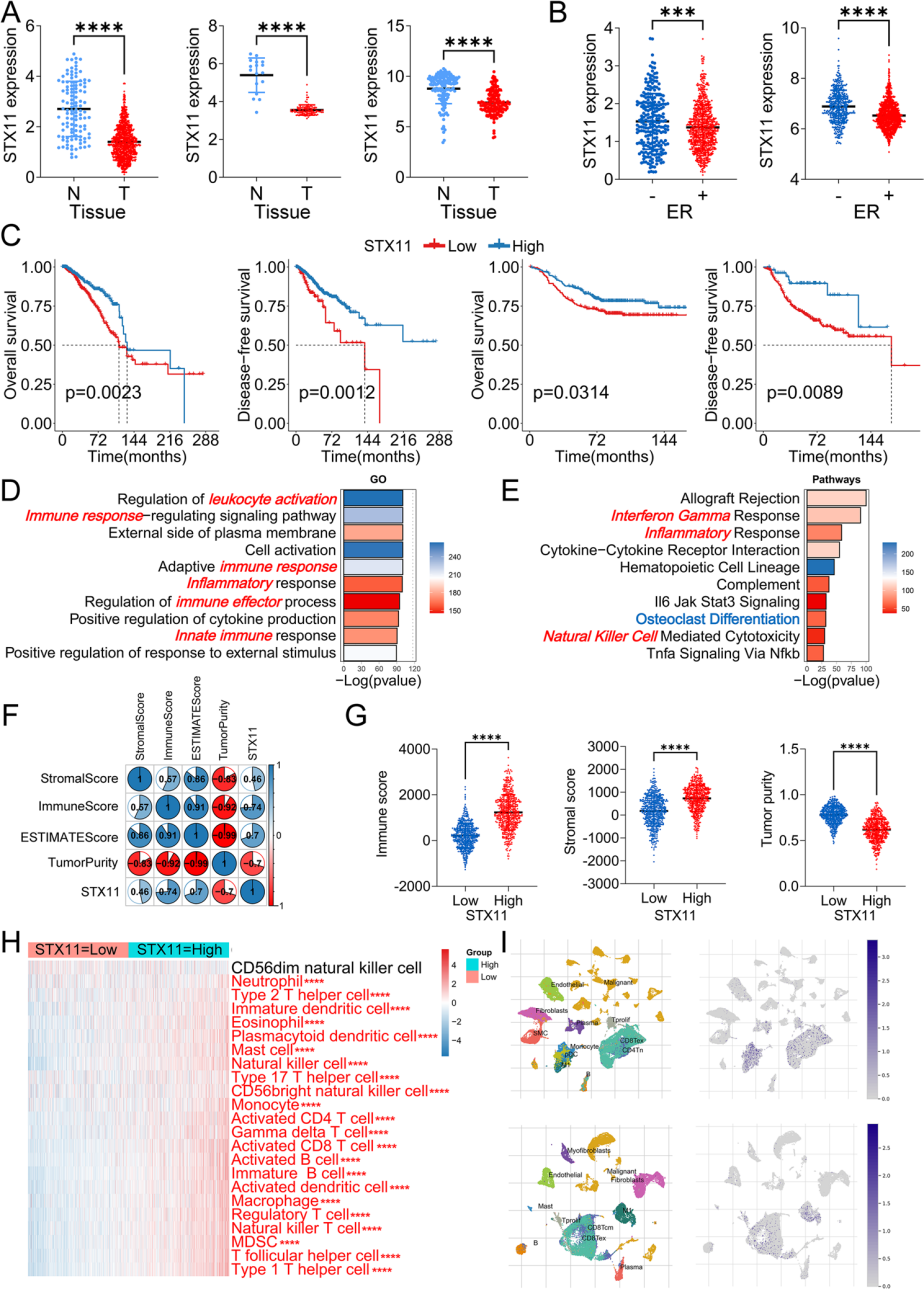
Bioinformatics analyses of STX11 in patients with BC. **A** Expression of STX11 between normal and BC tissues. **B** Expression of STX11 between ER positive and negative BC tissues. **C** Overall and disease-free survival curves between the low- and high-STX11 groups. **D** GO analyses of STX11-related genes. **E** Pathway analyses of STX11-related genes. **F** Correlation between the expression of STX11 and tumor microenvironment scores. **G** Immune scores, stromal scores and tumor purity between the low- and high-STX11 groups. **H** Immune cell infiltration between the low- and high-STX11 groups. **I** Single cell analyses showing STX11 expression profiles in BC samples; ****P<0.0001 **P<0.01 *P<0.05

We first analyzed the expression pattern of STX11 in BC. In addition to the TCGA cohort, we confirmed that STX11 expression was downregulated in tumor tissues in the GSE42568 and GSE70947 cohorts (Fig. 4A). Furthermore, the correlation between the expression levels of STX11 and ER was confirmed in both the TCGA and METABRIC datasets (Fig. 4B). However, the relationships between the expression levels of STX11 and PR and HER2 are not stable and still require further study. (Figure S4). To further determine the optimal expression cutoff of STX11 for diagnosing BC, an ROC curve of the TCGA cohort was generated. The AUC value was 0.837 (95% CI: 0.795–0.879), indicating favorable performance, and the optimal expression cutoff of STX11 was 2.280 (Figure S5). Survival analyses revealed that patients with BC with high STX11 expression had better outcomes in both the training and validation sets (Fig. 4C). In addition, STX11 expression was closely related to the distant metastasis of BC (Figure S6). To further elucidate the potential mechanism of STX11 in BC, correlation analyses were performed. A total of 1014 genes were confirmed as STX11-related genes, which were enriched in immune response functions and pathways, such as the inflammatory response, regulation of leukocyte activation, natural killer cell-mediated cytotoxicity, and TNF-α signaling via NF-κB (Fig. 4D and E). In addition, STX11-related genes were confirmed to be associated with osteoclast differentiation, a phenotype associated with bone metastasis, which may explain the association of STX11 with bone metastasis in BC (Fig. 4E and Figure S6). Overall, these results indicate that STX11 is potentially immunomodulatory in BC. Thus, we further analyzed the relationship between the tumor microenvironment and STX11 expression. The patients with BC with high STX11 expression presented higher immune and stromal scores and lower tumor purity (Fig. 4F and G). Specifically, all immune cells, except for CD56dim natural killer cells, exhibited significantly increased infiltration in patients with high STX11 expression (Fig. 4H). Previous studies have shown that STX11 plays an important role in macrophages (Zhang et al. 2008; Kinoshita et al. 2019). Therefore, we further analyzed its relationship with TAMs. Single-cell sequencing revealed that STX11 is expressed on immune cells, especially macrophages/myeloid cells (Fig. 4I and Figure S7A). Further analysis of myeloid cells revealed that STX11 was expressed at the highest level in M1 and FCN1 subtype cells, which are potential proinflammatory macrophage subtypes (Fig. 4I and Figure S7B). Data downloaded from xCell revealed that the expression of STX11 was positively correlated with the abundance of M1 macrophages (Figure S8). Correlation analyses revealed that the expression of STX11 was significantly associated with M1 macrophages (Figure S9). Therefore, although the expression of STX11 was positively correlated with the number of macrophages, the differential expression of STX11 in different subtypes of TAMs may be the key factor determining its correlation with the prognosis of BC patients. Overall, this study revealed that STX11 is potentially immunomodulatory in BC and may regulate the fate of macrophages to determine the prognosis of patients with BC.

### In vitro experiments confirmed that STX11 is a macrophage polarization regulator

**Fig. 5:**
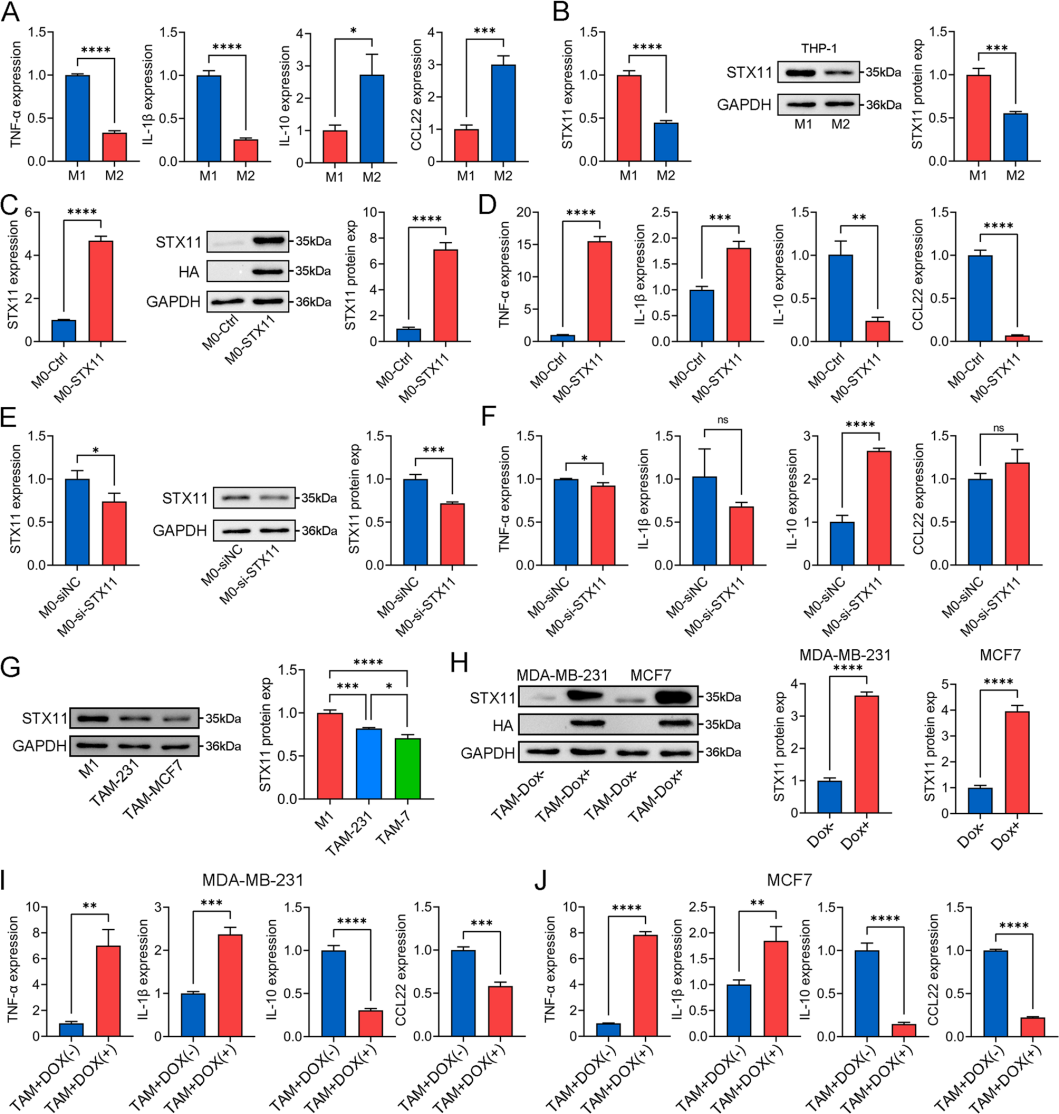
STX11 repolarized M1-like macrophages to the M1 phenotype.**A** Expression levels of TNF-α, IL-1β, IL-10 and CCL22 between M1-like and M2-like macrophages. **B** STX11 expression between M1-like macrophages and M2-like macrophages. **C** STX11 expression in M0-like macrophages after overexpression. **D** Expression profiles of genes associated with M1-like (TNF-α and IL-1β) and M2-like (IL-10 and CCL22) macrophages in M0-like macrophages after STX11 overexpression. **E** STX11 expression after siRNA treatment of M0-like macrophages. **F** Expression profiles of genes associated with M1-like (TNF-α and IL-1β) and M2-like (IL-10 and CCL22) macrophages in M0-like macrophages after STX11 silencing. **G** STX11 expression in LPS-induced M1-like macrophages and BC-induced TAMs. **H** STX11 expression in BC-induced TAMs after STX11 overexpression. **I** and **J** Expression profiles of genes associated with M1-like (TNF-α and IL-1β) and M2-like (IL-10 and CCL22) macrophages in BC-induced tumor-associated macrophages after STX11 overexpression

The role of STX11 in macrophage polarization was next confirmed. First, THP-1-derived macrophages were induced to differentiate into M1- or M2-like macrophages using LPS and IL-4, respectively. The qPCR results revealed that the markers of M1- or M2-like macrophages were significantly changed (Fig. 5A). STX11 expression was also confirmed to be lower in M2-like macrophages (Fig. 5B). STX11 was subsequently overexpressed in M0-like macrophages (Fig. 5C). Moreover, the expression levels of M1-like macrophage markers (TNF-α and Il-1β) were significantly increased in the STX11-overexpressing group, whereas the expression levels of M2-like macrophage markers (IL-10 and CCL22) were significantly decreased (Fig. 5D). In contrast, the expression levels of M1-like macrophages (TNF-α) and M2-type macrophage markers (IL-10) significantly changed after STX11 expression was silenced (Fig. 5E and 5F). Taken together, these results suggest that STX11 can promote the M1 polarization and inhibit the M2 polarization of macrophages.

We further investigated the effect of STX11 on repolarized tumor-associated macrophages (TAMs). First, we silenced the expression of STX11 in LPS-induced M1-like macrophages, and the results indicated that the expression levels of M1-like macrophage markers (TNF-α and Il-1β) were significantly decreased, whereas the expression levels of M2-like macrophage markers (IL-10 and CCL22) were significantly increased (Figure S10). Finally, THP-1-derived M0-like macrophages were cocultured with BC cells to generate TAMs. These TAMs presented increased expression of M2-like macrophage markers (IL-10 and CCL22), whereas the expression of STX11 was inhibited (Figure S11). After STX11 was overexpressed in TAMs, the phenotype of these macrophages was successfully repolarized to that of M1-like macrophages (Fig. 5G-5J). In summary, these results indicate that STX11 not only promotes the differentiation of M0 macrophages into M1 macrophages but also effectively converts TAMs into M1 macrophages.

### STX11-overexpressing macrophages inhibit the malignancy of BC cells

**Fig. 6:**
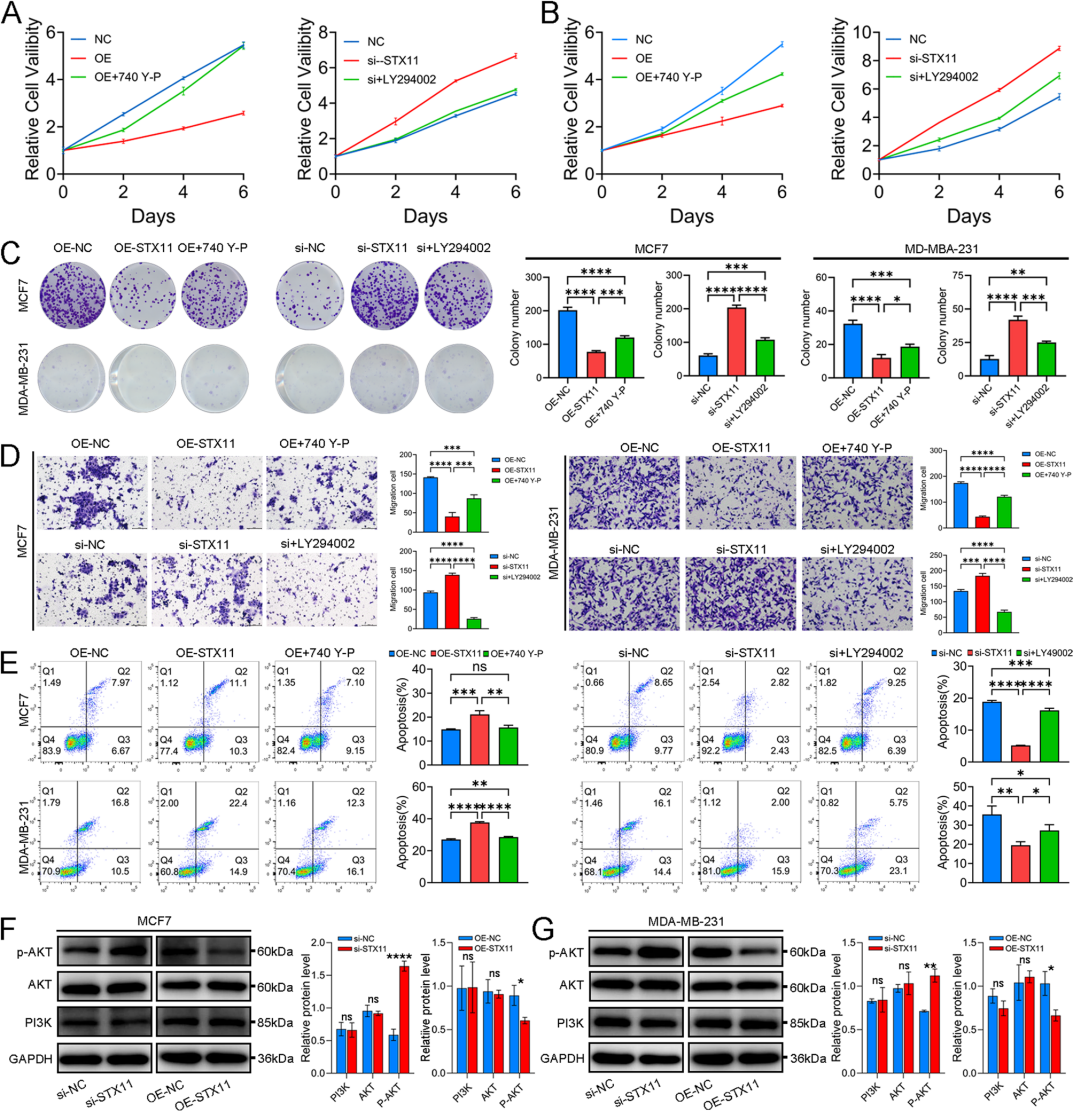
The effect of STX11 expression in macrophages on BC malignancy was studied *in vitro*. **A** CCK-8 assays of MCF7 cells after coculture with THP-1-derived macrophages. **B** CCK-8 assays of MDA-MB-231 cells after coculture with THP-1-derived macrophages. **C** Colony formation of MCF7 and MDA-MB-231 cells after coculture with THP-1-derived macrophages. **D** Transwell assays of MCF7 and MDA-MB-231 cells after coculture with THP-1-derived macrophages. **E** Flow cytometry of apoptosis in MCF7 and MDA-MB-231 cells after culture with THP-1-derived macrophages. **F** Western blotting of PI3K–AKT proteins in MDA-MB-231 cells after coculture with THP-1-derived macrophages with STX11 knockdown or overexpression. **G** Western blotting of PI3K–AKT proteins in MCF7 cells after coculture with THP-1-derived macrophages with STX11 knockdown or overexpression

In previous studies, we confirmed the role of STX11 in reprogrammed macrophage polarization, and bioinformatics analyses indicated that patients with BC with high STX11 expression presented favorable outcomes. According to the literature, STX11 is a SNARE family member closely related to the PI3K/AKT pathway (Huang et al. 2024). This pathway is closely related to BC malignancy. Thus, we further tested whether STX11-overexpressing or STX11-silenced macrophages affect the malignancy and PI3K/AKT pathway of BC cells. A CCK-8 assay revealed that STX11-overexpressing macrophages effectively inhibited the proliferation of MCF-7 and MDA-MB-231 cells, whereas macrophages with silenced STX11 promoted the proliferation of MCF-7 and MDA-MB-231 cells (Fig. 6A and B). The results of the colony formation assays were consistent with those of the CCK8 assay (Fig. 6C). Transwell assays revealed that STX11-overexpressing macrophages effectively inhibited the migration of MCF-7 and MDA-MB-231 cells, whereas macrophages with silenced STX11 promoted the migration of both cell lines (Fig. 6D). Flow cytometry revealed that STX11-overexpressing macrophages effectively promoted the apoptosis of MCF-7 and MDA-MB-231 cells, whereas STX11-silenced macrophages had the opposite effect (Fig. 6E). Finally, the expression levels of proteins related to the PI3K–AKT signaling pathway were verified. The activity of the PI3K–AKT signaling pathway was negatively correlated with the expression levels of STX11 in both MCF7 and MDA-MB-231 cells (Fig. 6F and G). To further confirm the role of the PI3K–AKT signaling pathway in BC cells, 740Y-P and LY294002 were used to intervene in this pathway. The results revealed that the malignant phenotype of BC cells caused by STX11 overexpression in macrophages was significantly reversed (Fig. 6A and E). This result was also confirmed by silencing STX11 (Fig. 6A and E). Overall, our results suggest that STX11 can promote macrophage M1 polarization and inhibit the PI3K–AKT signaling pathway to promote BC cell apoptosis.

### In vivo experiments confirmed that STX11 is a tumor suppressor in BC

**Fig. 7:**
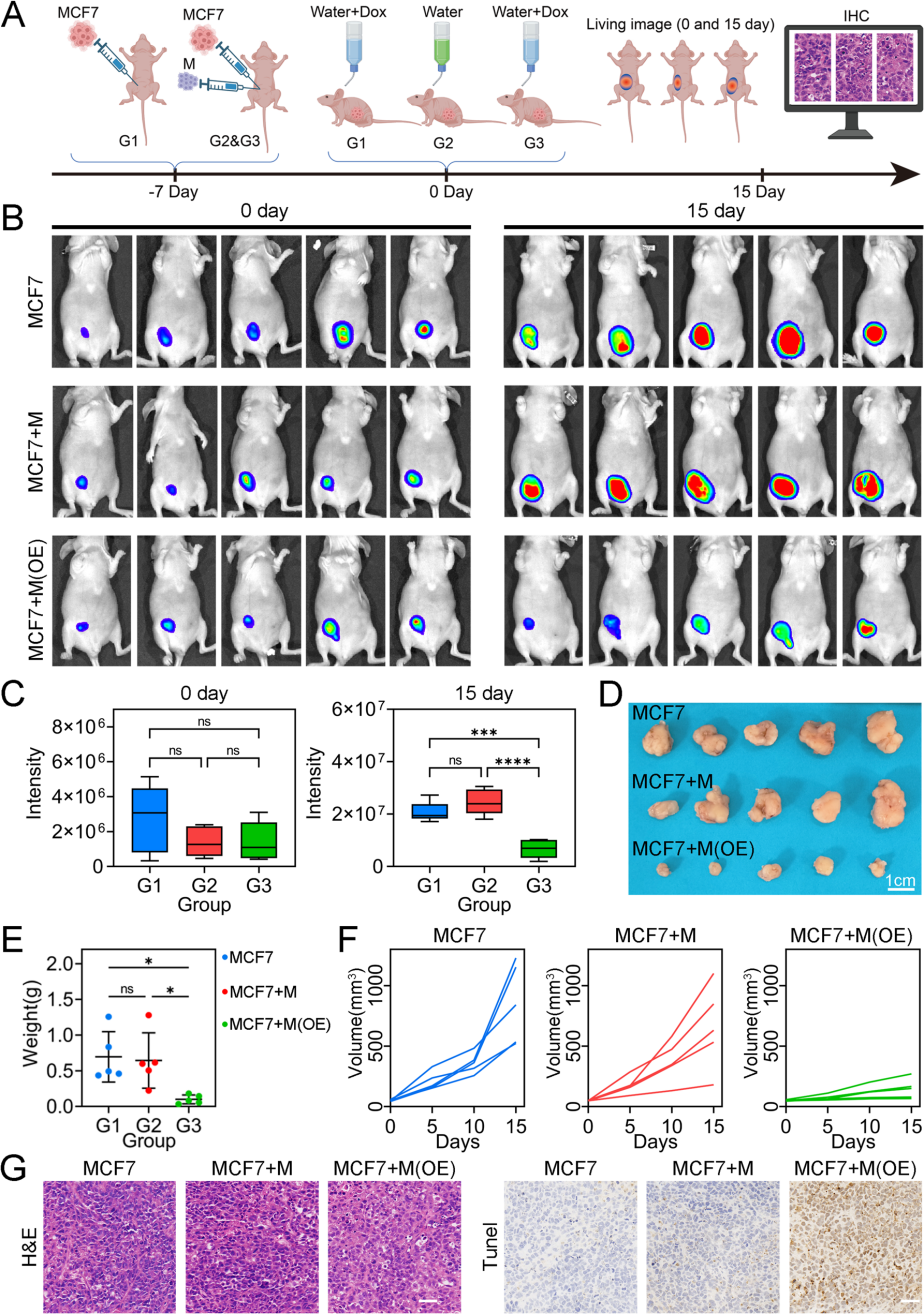
STX11 overexpression in macrophages inhibits primary BC *in vivo*. **A** The procedure of *in vivo* experiments. **B** Results of live imaging of three groups of mice. **C** Comparison of fluorescence intensity in live imaging of mice at 0 and 15 days. **D** Subcutaneous BCs surgically removed from each group. **E** Average tumor weights in each group of BC models. **F** Spider diagrams showing the growth kinetics of the individual tumor volume of the BC model. **G** Hematoxylin‒eosin (**H**&**E**) and immunohistochemical (IHC) staining of tumor tissues

We further explored the role of STX11 in vivo (Fig. 7A). After THP-1-derived macrophages and/or MCF-7 cells were injected, there was no significant difference in tumor volume among the three groups before they were fed Dox to interfere with STX11 expression (Fig. 7B and C). STX11 was then overexpressed in macrophages after Dox feeding, which effectively inhibited the proliferation of BC (Fig. 7B and C). The tumor volumes in the MCF-7 and MCF-7 + macrophage groups were significantly greater than that in the MCF7 + macrophage (STX11-overexpressing) group (Fig. 7D and E), and the weight of the tumors in this group was also the lowest (Fig. 7F). Finally, we performed histological staining, and the IHC results revealed that the cells in the MCF7 + macrophage (STX11 overexpression) group had the deepest TUNEL staining, indicating the highest apoptosis rate (Fig. 7G). In summary, the in vivo results indicate that macrophages overexpressing STX11 are anticancer immune cells that can effectively inhibit the proliferation of MCF-7 cells.

### Pancancer analysis confirmed STX11 expression is closely related to the malignant characteristics of several tumors

**Fig. 8:**
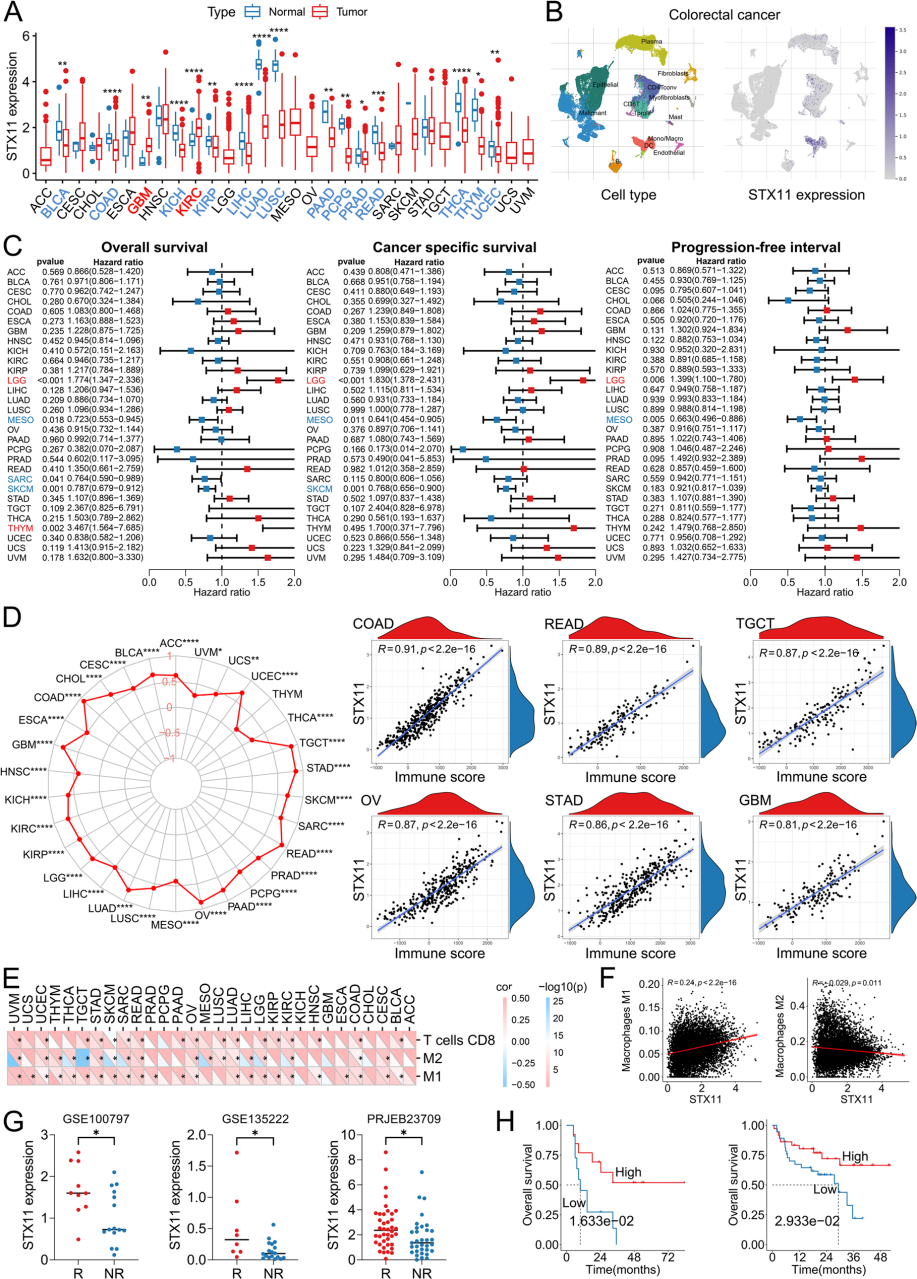
Pancancer analyses of STX11.**A** Expression of STX11 in normal and tumor tissues. **B** STX11 expression in colorectal cancer tissue based on single-cell sequencing analysis. **C** Overall survival, cancer-specific survival and progression-free interval analyses of STX11. **D** Correlations between the expression of STX11 and the immune score across 30 cancers. **E** Correlations between the expression of STX11 and typical immune cells across 30 cancers.**F** Correlations between STX11 expression and macrophages in the pancancer cohort. **G** STX11 expression between patients with cancer who were responsive and nonresponsive to immunotherapy.**H** Survival curve of patients with cancer who received immunotherapy.

In the pancancer cohort, STX11 was downregulated in 14 of the 30 solid tumors (Fig. 8A). Consistent with the results from the BC datasets, the results of single-cell RNA sequencing revealed that STX11 was expressed mainly in macrophages in a variety of tumors but was rarely expressed in tumor cells (Fig. 8B and Figure S12-S19). Survival analysis revealed that STX11 plays different roles in different tumors. For example, patients with LGG with high STX11 expression have a poor prognosis, whereas patients with MESO with high STX11 expression have a favorable prognosis (Fig. 8C). We further analyzed the relationship between STX11 expression and the TME. Surprisingly, STX11 expression was positively correlated with the immune scores of all 30 tumors (Fig. 8D). Specifically, for M1 macrophages, M2 macrophages and CD8+ T cells, the infiltration of CD8+ T cells increased with increasing STX11 expression in 17 tumors, which was also consistent with the results from the BC datasets (Fig. 8E). In experimental studies, we demonstrated that STX11 overexpression promotes M1 polarization of macrophages. In 21 tumors in the pancancer cohort, STX11 expression was positively correlated with the number of M1 macrophages and negatively correlated with the number of M2 macrophages in 9 tumors (Fig. 8E). A combination of data from 30 tumors for analysis revealed that STX11 expression was positively correlated with the number of M1 macrophages and negatively correlated with the number of M2 macrophages at the pancancer level (Fig. 8F). These results revealed that STX11 is a conserved regulator of macrophage polarization across solid tumors. Finally, we analyzed the effects of STX11 in a real immunotherapy cohort. The results revealed that STX11 expression was significantly higher in patients who responded to immunotherapy than in patients who did not respond to immunotherapy (Fig. 8G). Patients with high STX11 expression had a better prognosis after treatment with ICIs (Fig. 8H).

## Discussion

In this study, we conducted a systematic bioinformatics analysis using RNA sequencing data from patients with BC to establish two prognostic models that can predict OS and DFS. Both signatures showed powerful prognostic prediction ability and were validated in independent datasets. In addition, STX11 was included in both the OS and DFS prognostic signatures and was expressed mainly in macrophages in the TME. Silencing and overexpression experiments confirmed that STX11 is a polarizing promoter of M1 macrophages and can regulate the malignancy of BC cells by regulating the PI3K–AKT signaling pathway. In vivo experiments confirmed that STX11 overexpression in TAMs effectively inhibited BC progression.

The relationship between HPV and BC is still unclear, and its relationship with the TME has been less well reported. In our research, both the HPV-based signature and the vital gene STX11 were closely related to the prognosis and TME of patients with BC. However, the HPV-based signature has a poor prognostic predictive effect on the basal subtype, which might be due to the relatively small number of patients with this subtype in our study. The carcinogenic factors of different subtypes of BC also vary significantly, and whether HPV or HPV-related genes play different roles in these cancers still requires further research (Heger et al. 2024; Onkar et al. 2023). Currently, no studies have reported the relationship between HPV and the TME in BC, but this relationship has been extensively confirmed in other tumors. There are significant differences in the TME, including tumor stemness and lymphocyte infiltration, between HPV-positive and HPV-negative tumors, and the presence of HPV-specific T-cell responses has been confirmed in these tumors (Lin et al. 2023; Ji et al. 2023; Cao et al. 2023; Cheng et al. 2023). In addition, in HPV-associated oral cancer, there is a CD161^+^ cytotoxic T lymphocyte (CTL) subpopulation with both activation and exhaustion phenotypes, and its production is closely related to HPV infection (Wei et al. 2023). These findings suggest that HPV can substantially impact the remodeling of the TME. Although we have not directly confirmed that HPV infection is related to the TME of BC, we believe that there is a potential relationship between HPV infection and macrophages in BC through bioinformatics analysis of the HPV-related gene STX11 and validation in vivo and in vitro. In the future, it will be necessary to study how HPV regulates the gene expression patterns of BC cells and the remodeling of the TME, which will help in the development of treatments against HPV and help in the development of precision treatments for HPV-positive patients.

In this study, STX11 was confirmed to be a novel macrophage polarization regulator that promotes macrophage M1 polarization and plays an anticancer role. However, only a few studies have focused on the role of STX11 in solid tumors, and it has been identified as a tumor suppressor in T-cell lymphomas; yet, the mechanism is still unclear (Yoshida et al. 2014, 2015). We confirmed that STX11-overexpressing macrophages can inhibit the malignancy of BC cells by coculture, suggesting that these cells secrete active substances to regulate the fate of tumor cells. With the development of single-cell sequencing technology, TAMs can be further subdivided into various subtypes based on their gene expression patterns, and some subtypes can exert anticancer effects through secretion, which provides a theoretical basis for developing novel treatment strategies by interfering with polarization regulators of TAMs (Zhang et al. 2024). In our study, macrophages overexpressing STX11 (a macrophage polarization regulator) exhibited proinflammatory effects. Through macrophage-BC cell coculture, we confirmed that these compounds could effectively inhibit the proliferation and migration of tumor cells through secretion, but the mechanism is still unclear. In addition, TAMs can regulate CD8 + T-cell depletion by secreting exosomes in other ways (Pu et al. 2021). However, we did not study the relationship between STX11 and CD8 + T cells in this study, which will require further research in the future. Therefore, further elucidation of the mechanism by which STX11 regulates M1 polarization of macrophages will be necessary in the future.

In addition to its role in BC, we also analyzed the role of STX11 in a pancancer cohort. Surprisingly, STX11 was confirmed to be a potential pancancer regulator of M1 macrophage polarization. STX11 expression was positively correlated with the number of M1 macrophages and negatively correlated with the number of M2 macrophages in many tumors. However, STX11 has been shown to be correlated with prognosis in only a few tumors. Interestingly, STX11 was positively correlated with M1 macrophages and negatively correlated with M2 macrophages in LGG, but its relationship with prognosis was opposite to that in BC. Although the traditional M1 and M2 classifications are considered to be related to the prognosis of patients with gliomas, recent single-cell sequencing revealed that glioma-associated macrophages can be further classified more precisely, such as AIF1^+^TAMs, SPP1^+^TAMs and CCL3^+^TAMs^49^. Furthermore, the composition of the glioma microenvironment is rather complex. Both myeloid cells and T cells are closely related to tumor malignancy (Barakat et al. 2024; Rajendran et al. 2023). Therefore, the role of STX11 in LGG may require more precise tools, such as single-cell sequencing analysis, spatial transcriptome sequencing, or protein sequencing, for in-depth study. In previous studies, the relationships between STX11 and both lung adenocarcinoma and colorectal cancer have been reported, but the relationships are still unclear (Zhu et al. 2023; Shen et al. 2020). Although STX11 is a SNARE protein that mediates the fusion of cytotoxic granules with the plasma membrane at the immunological synapses of CD8 + T or NK cells, the role of this gene in macrophage polarization is unclear (Kögl et al. 2024). Further research on how STX11 regulates macrophage polarization will help promote further clinical applications.

Although we confirmed that the HPV-associated gene STX11 can play an anticancer role by regulating the polarization of tumor-associated macrophages, there are still shortcomings in this study. First, as a macrophage M1 polarization regulator, the polarization mechanism of STX11 and its function in other immune cells were not thoroughly evaluated. Second, although we established and validated two signatures for predicting the OS and DFS of patients with BC, their performance needs to be further verified with more external data. Third, our study screened STX11 in BC samples with or without HPV infection, but the interaction between them was not revealed in this study, which is also one of the key research directions for the future. Finally, although we verified the function of STX11 through in vivo and in vitro methods, real-world clinical specimen verification, which is highly valuable, is lacking.

## Conclusion

In this study, we developed two prognostic signatures based on HPV-associated genes to predict the OS and DFS of patients with BC. HPV-related signatures were related to the prognosis, immune microenvironment and immunotherapy response of patients with BC. In addition, STX11 was demonstrated to be a tumor suppressor that inhibits the PI3K–AKT signaling pathway in BC cells to determine malignancy by regulating macrophage polarization.

## Supplementary Information

Supplementary Material 1.

## Data Availability

The data are available from the corresponding author on reasonable request.

## Abbreviations

HPV: Human papillomavirus
TME: Tumor microenvironment
BC: Breast cancer
TAM: Tumor-associated macrophage
GEO: Gene expression omnibus
OS: Overall survival
DFS: Disease-free survival
CAHG: Cancer-related HPV-related gene
PPI: Protein-protein interaction
GO: Gene Ontology
KEGG: Kyoto Encyclopedia of Genes and Genomes
K-M: Kaplan-Meier
ROC: Kaplan-Meier
AUC: Area under the curve
ssGSEA: Single sample gene set enrichment analysis
GSVA: Gene set variation analysis
MFS: Metastasis-free survival
BMFS: Bone metastasis-free survival
siRNA: small-interfering RNA
CCK8: Cell Counting Kit-8
RIPA: Radioimmunoprecipitation assay
IHC: Immunohistochemistry
EMT: Epithelial-mesenchymal transition
CTL: Cytotoxic T lymphocyte
