# Supplementary material for: Human papillomavirus-related syntaxin 11 reprograms tumor-associated macrophages to induce breast cancer cell apoptosis via PI3K/AKT signaling
Source: Mol Med. 2025 Sep 2;31:285. doi: 10.1186/s10020-025-01325-z (PMC12403928; doi:10.1186/s10020-025-01325-z)
Supplement: Supplementary file 1 — Supplementary Material 1. [file 10020_2025_1325_MOESM1_ESM.docx]

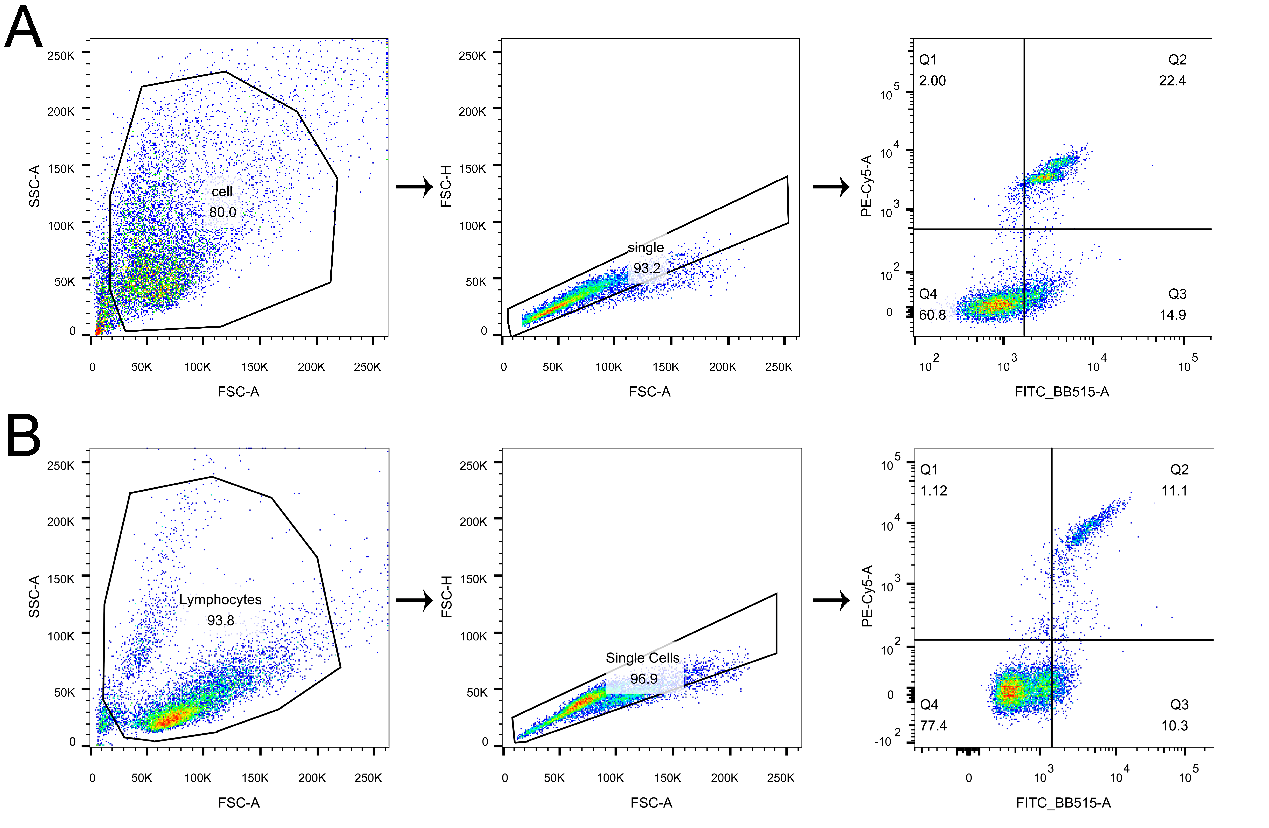


**Figure S1**. Gating strategy for flow cytometry in MDA-MB-231 (A) and MCF7 (B)


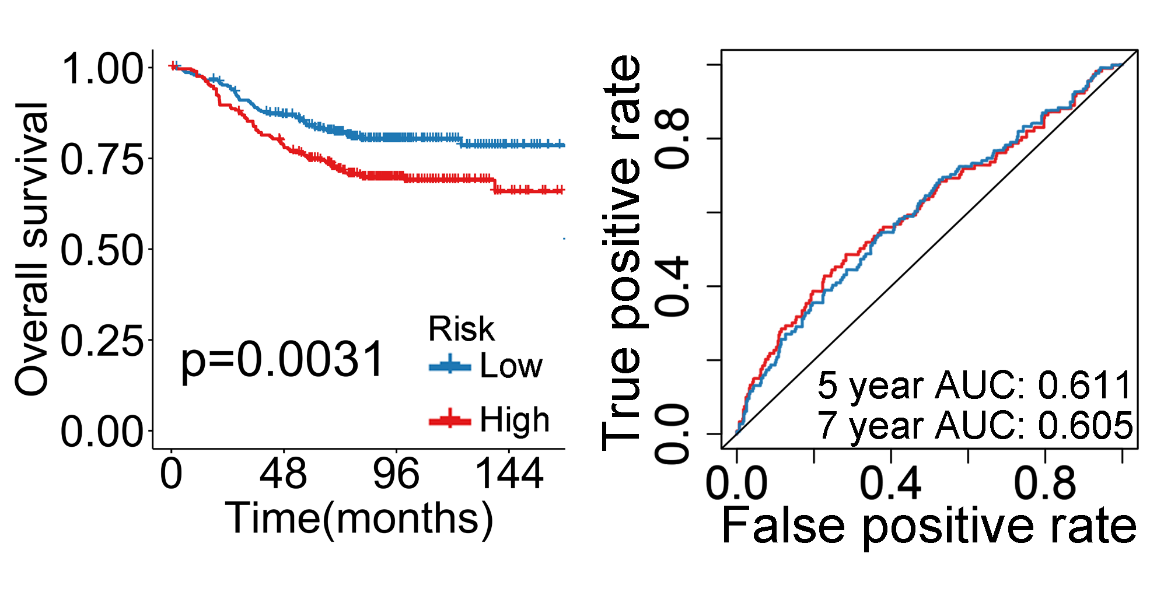


**Figure S2**. Survival and time-dependent ROC curves of OS signature in validation cohort


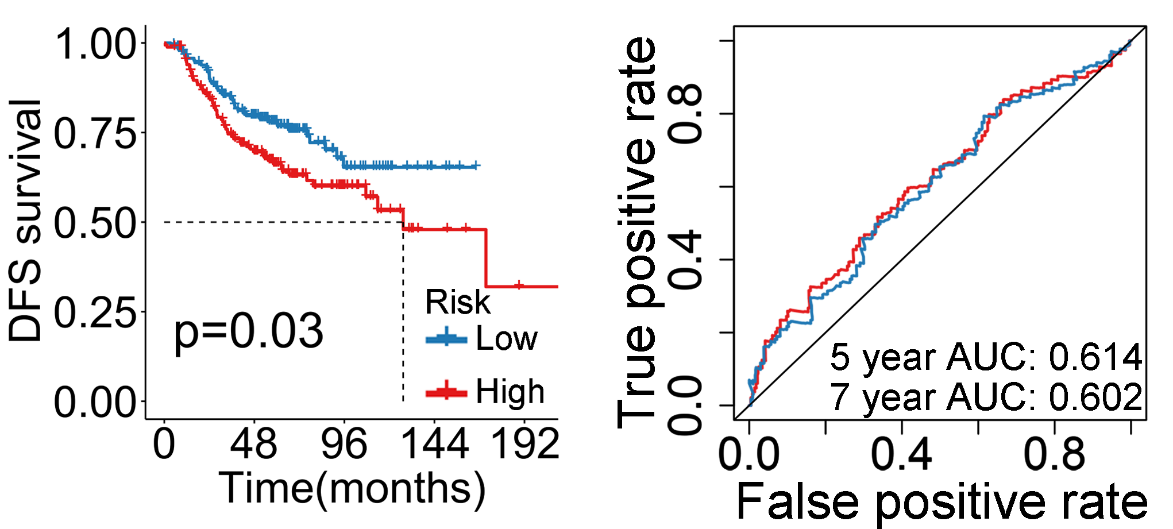


**Figure S3**. Survival and time-dependent ROC curves of DFS signature in validation cohort


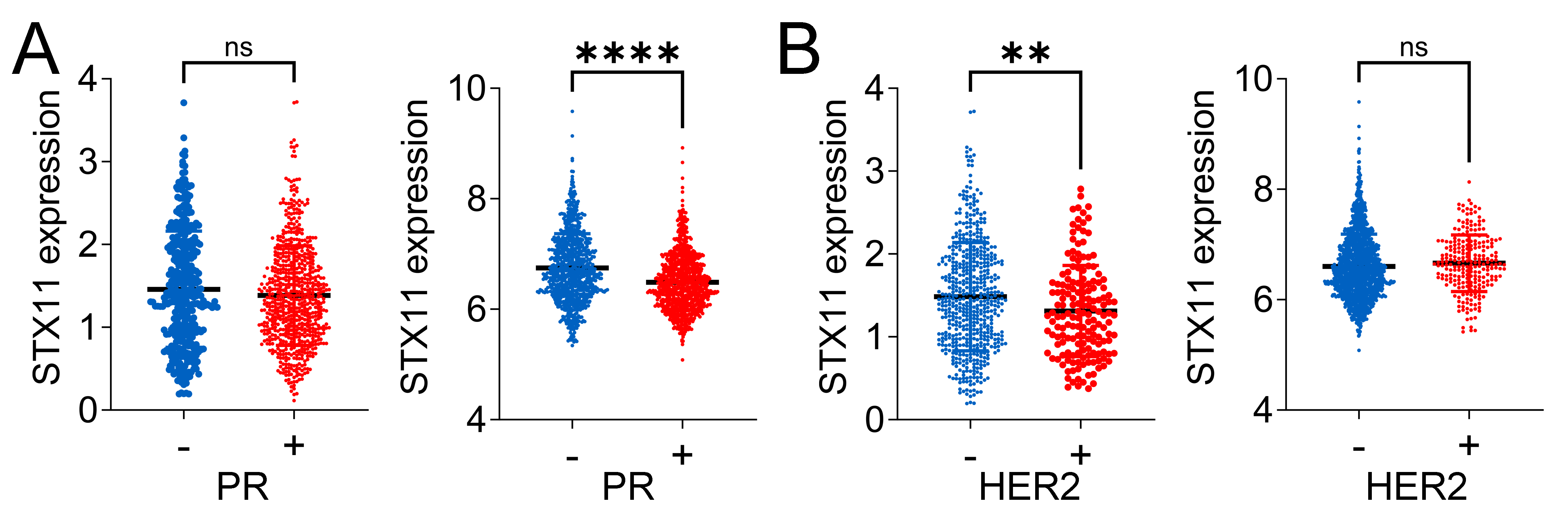


**Figure S4**. Expression of STX11 in breast cancer tissues


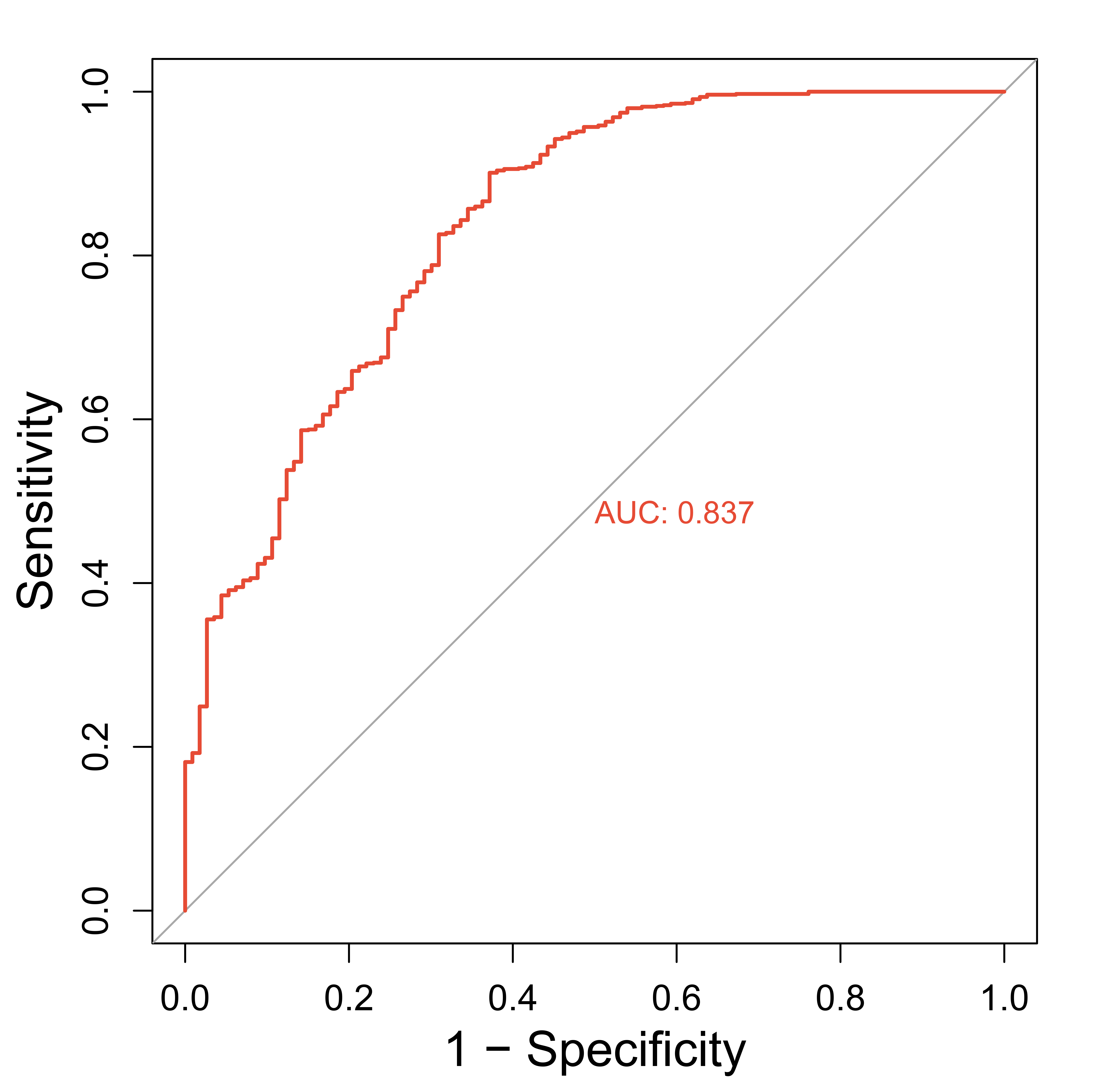


**Figure S5**. ROC curve of STX11 in diagnosing BC


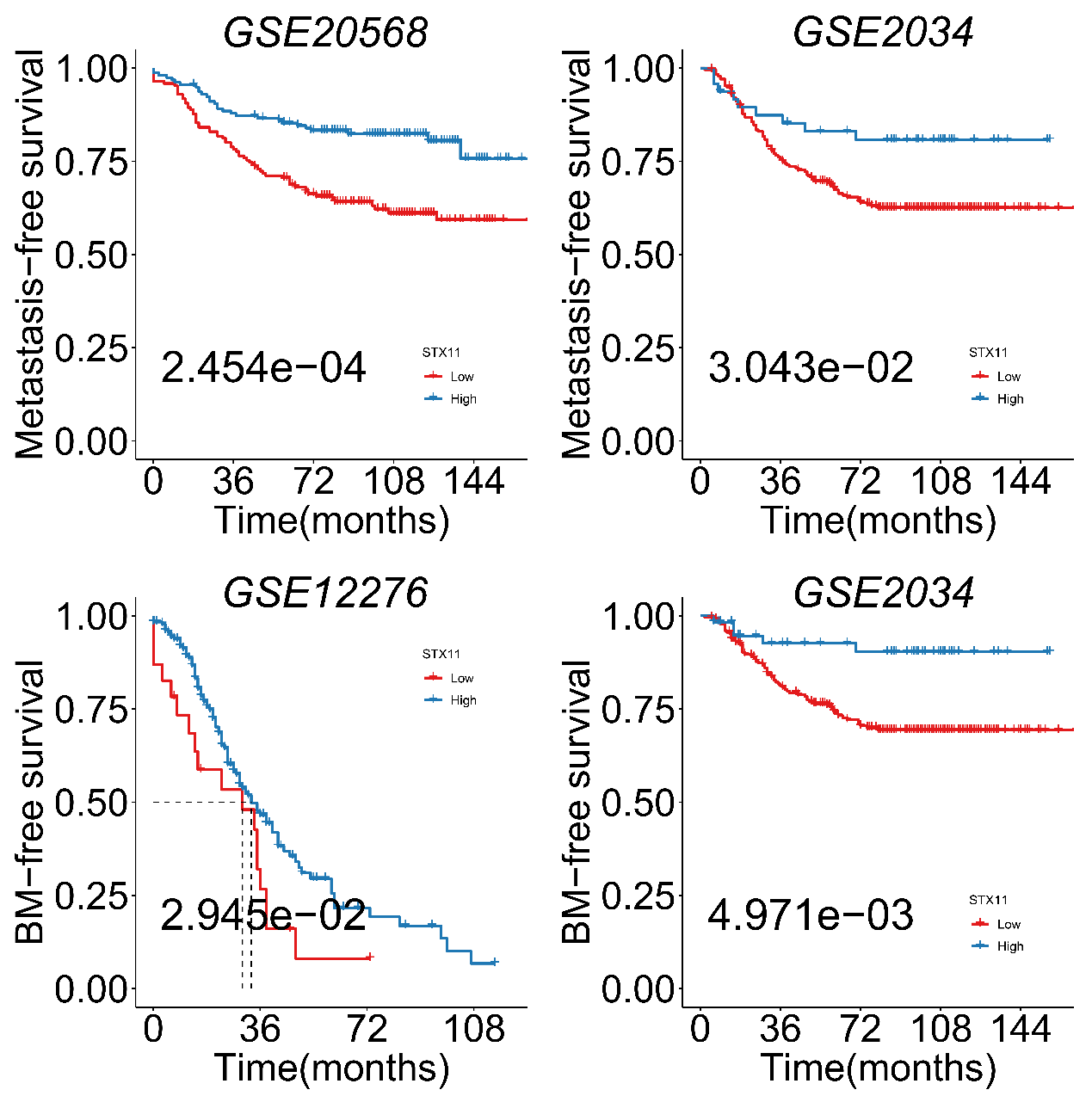


**Figure S6**. Survival curves of breast cancer patients with low- and high-STX11 expression


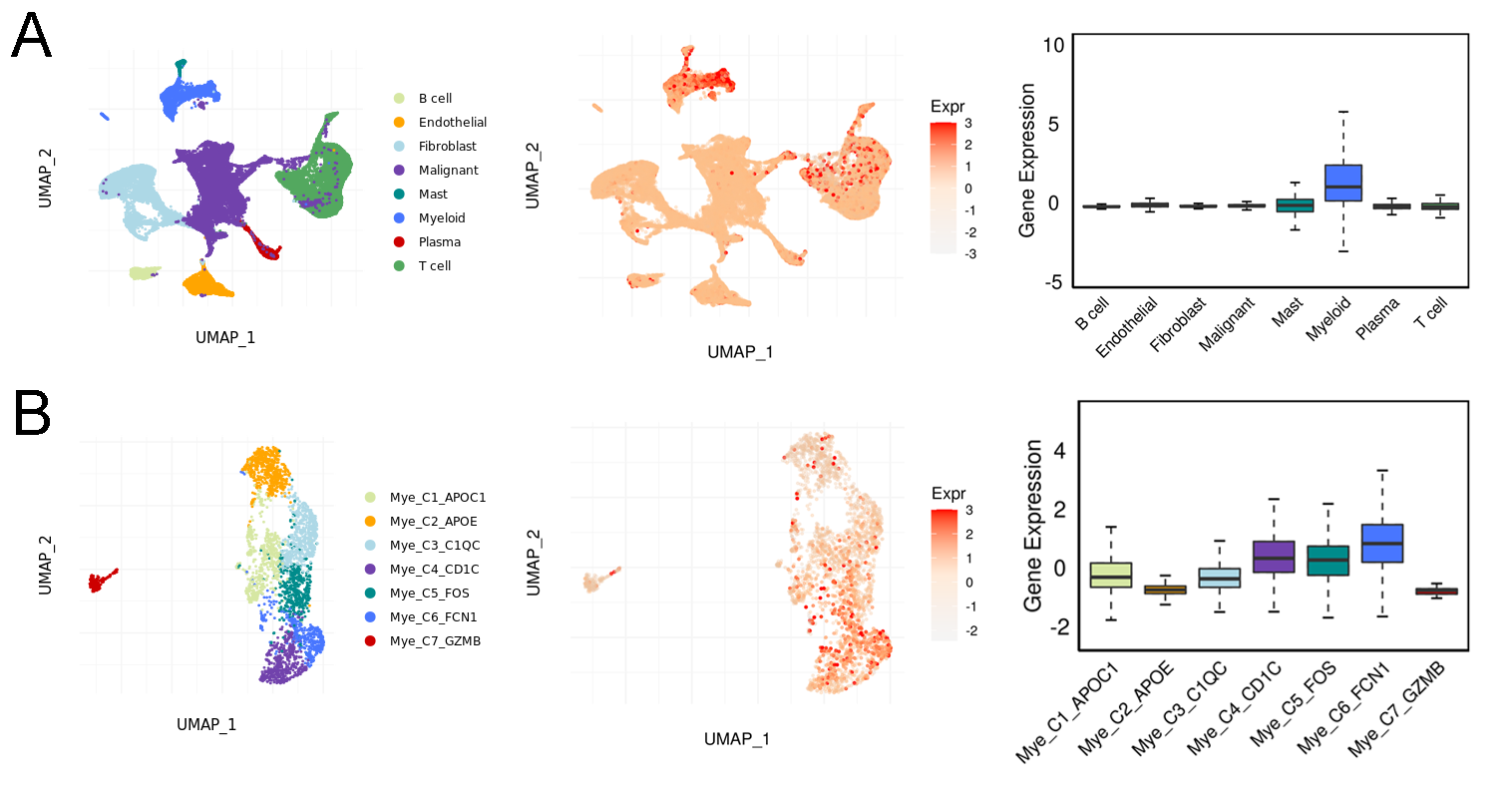


**Figure S7**. Single cell analyses of STX11 in BC tissues


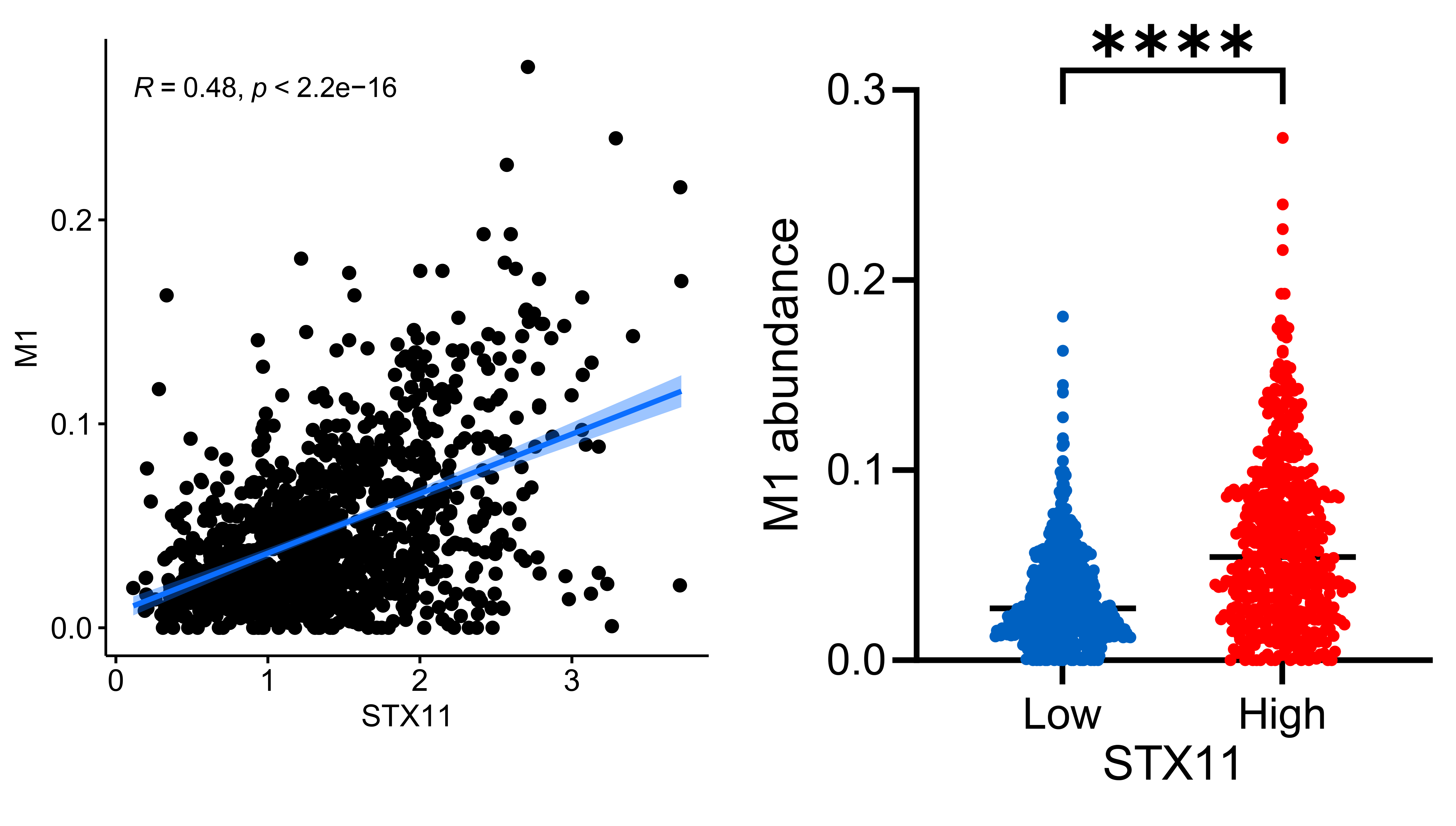


**Figure S8**. Correlation analyses of STX11 and M1 macrophage based on xCell.


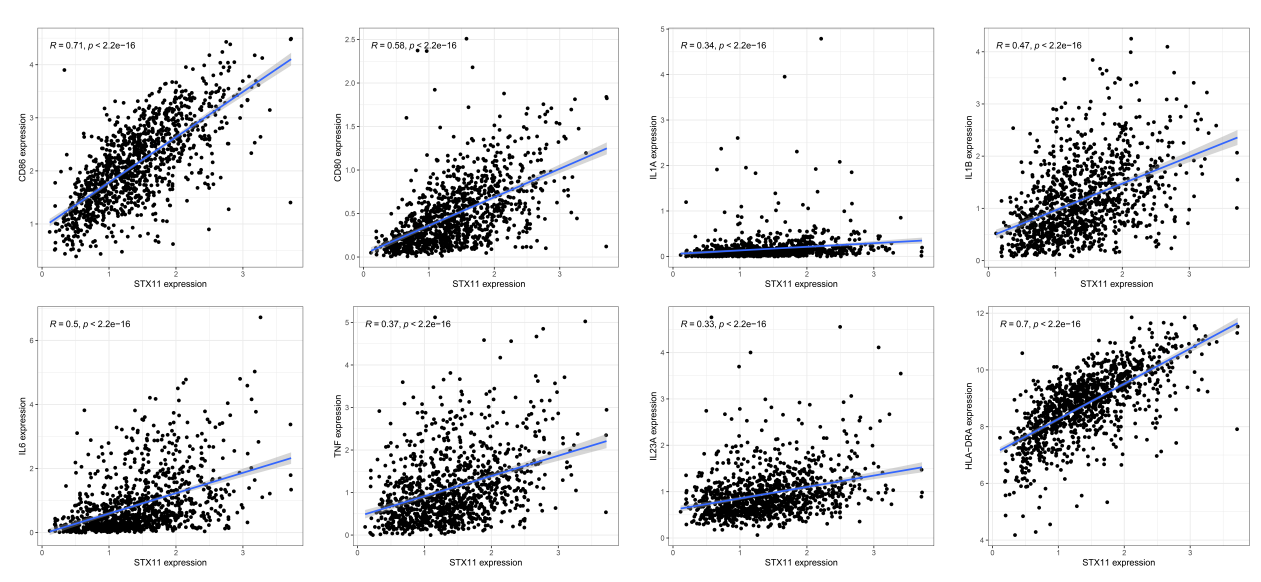


**Figure S9**. Correlation analyses of STX11 and macrophage markers.


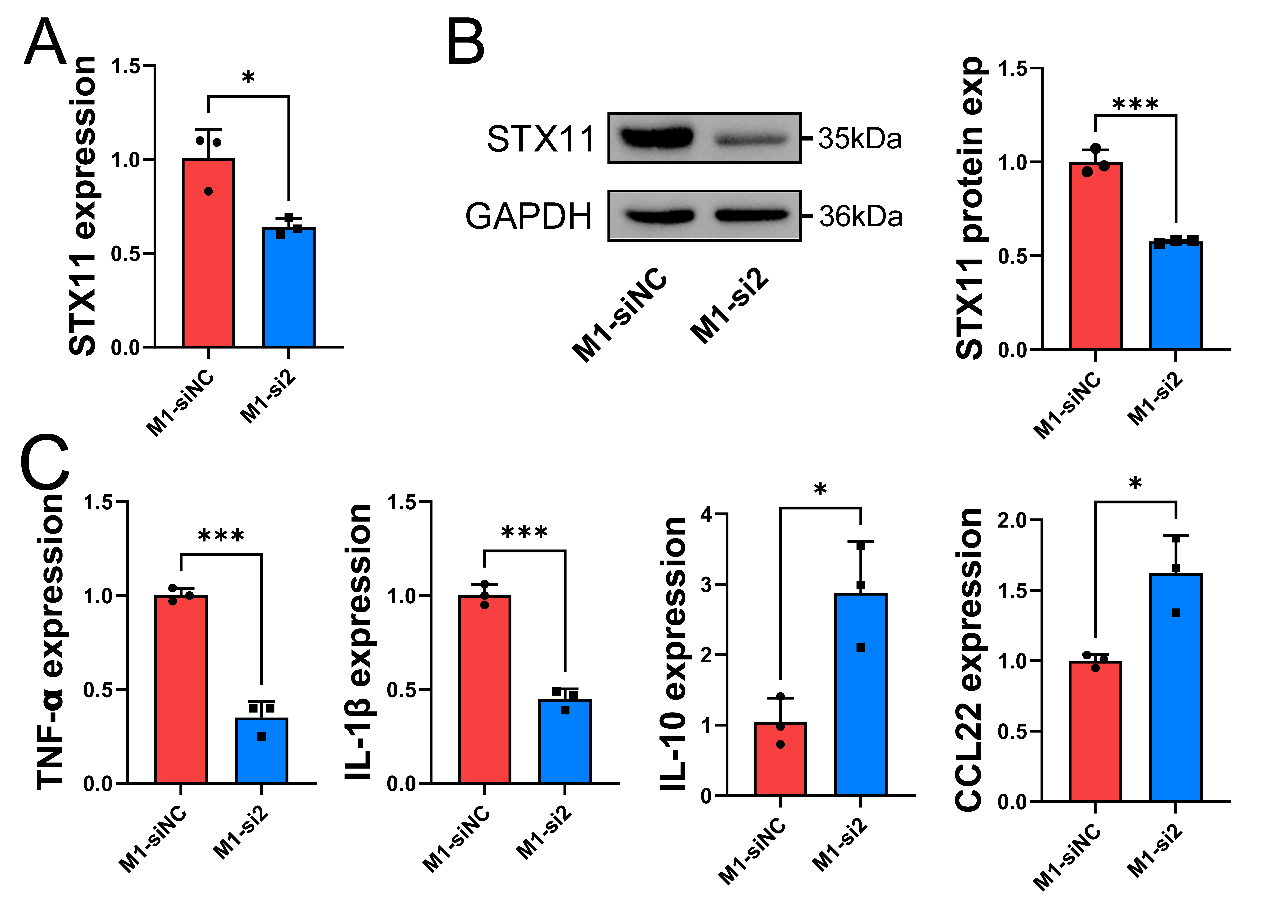


**Figure S10**. (A) The mRNA expression of STX11 after siRNA treated in M1-like macrophage; (B) The protein expression of STX11 after siRNA treated in M1-like macrophage; (C) The expression of genes associated with M1-like (TNF-α and IL-1β) and M2-like (IL-10 and CCL22) macrophages in M0-like macrophage after STX11 silencing


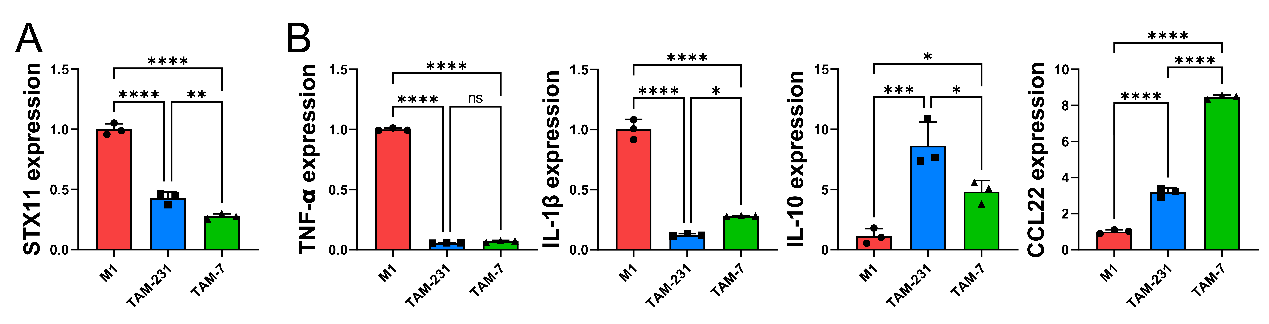


**Figure S11**. Expression of STX11(A) and genes associated with M1-like (TNF-α and IL-1β) and M2-like (IL-10 and CCL22) macrophages in tumor-associated macrophage (TAM) (B).


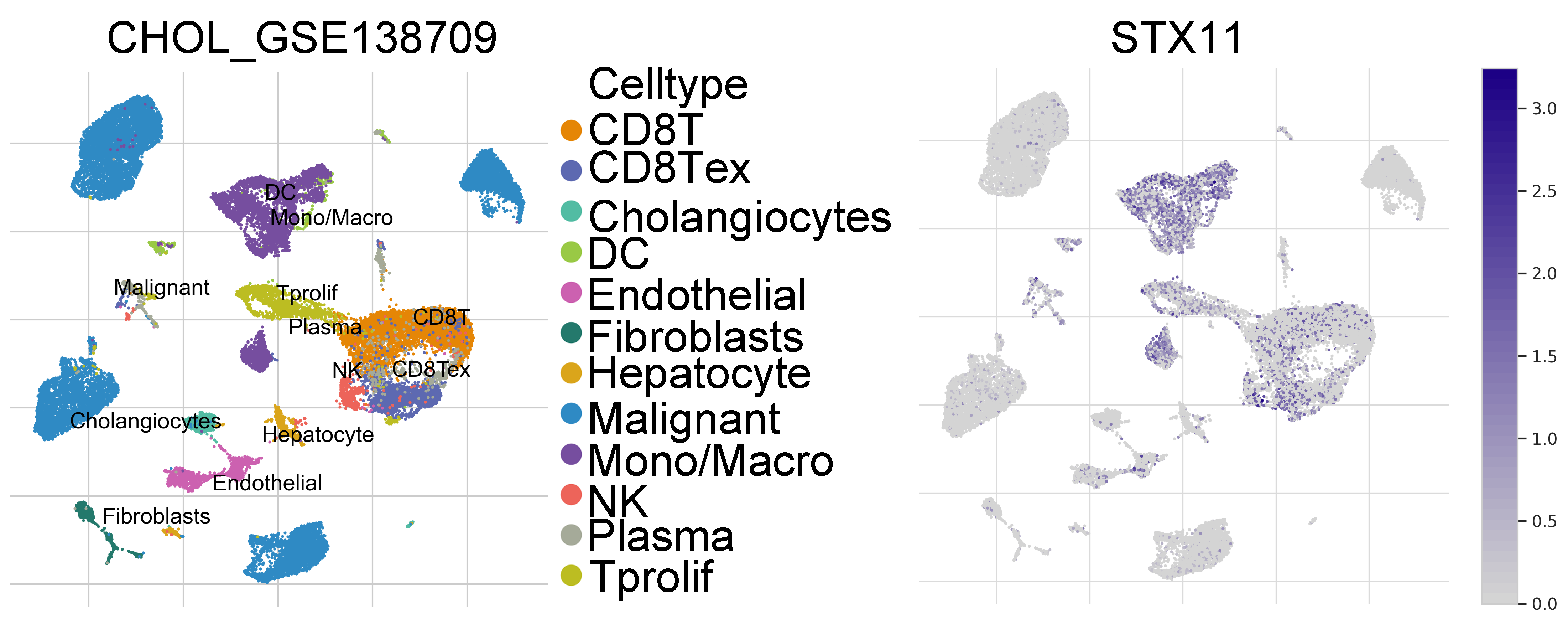


**Figure S12**. Single cell analysis of STX11 in cholangicarcinoma.


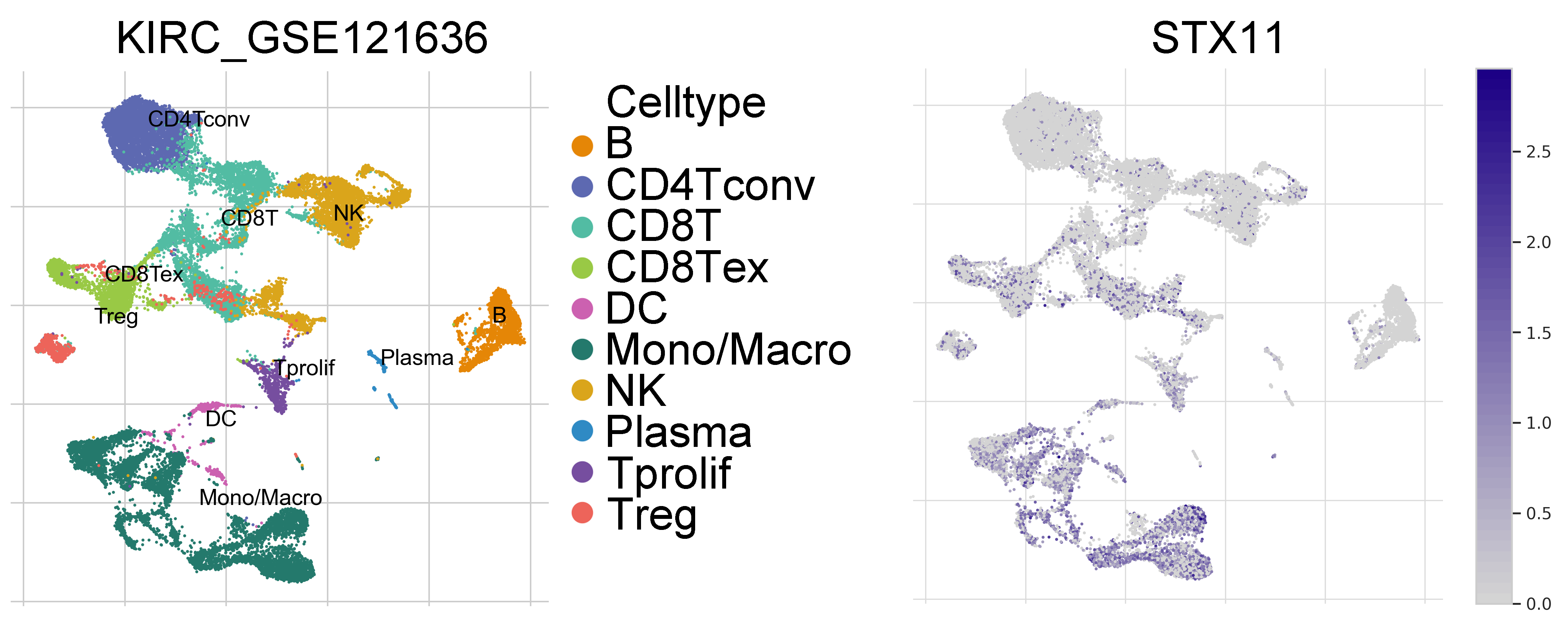
**Figure S13**. Single cell analysis of STX11 in kidney clear cell carcinoma.


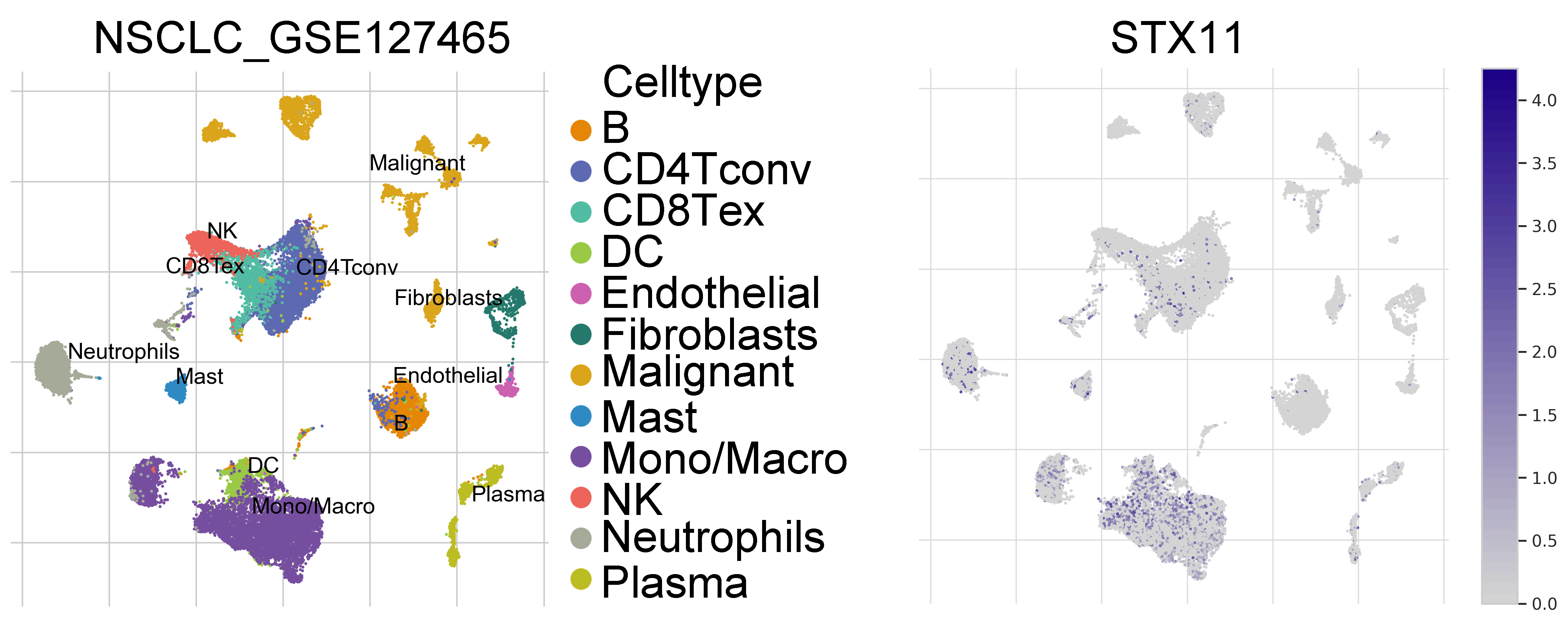
**Figure S14**. Single cell analysis of STX11 in non-small cell lung cancer.


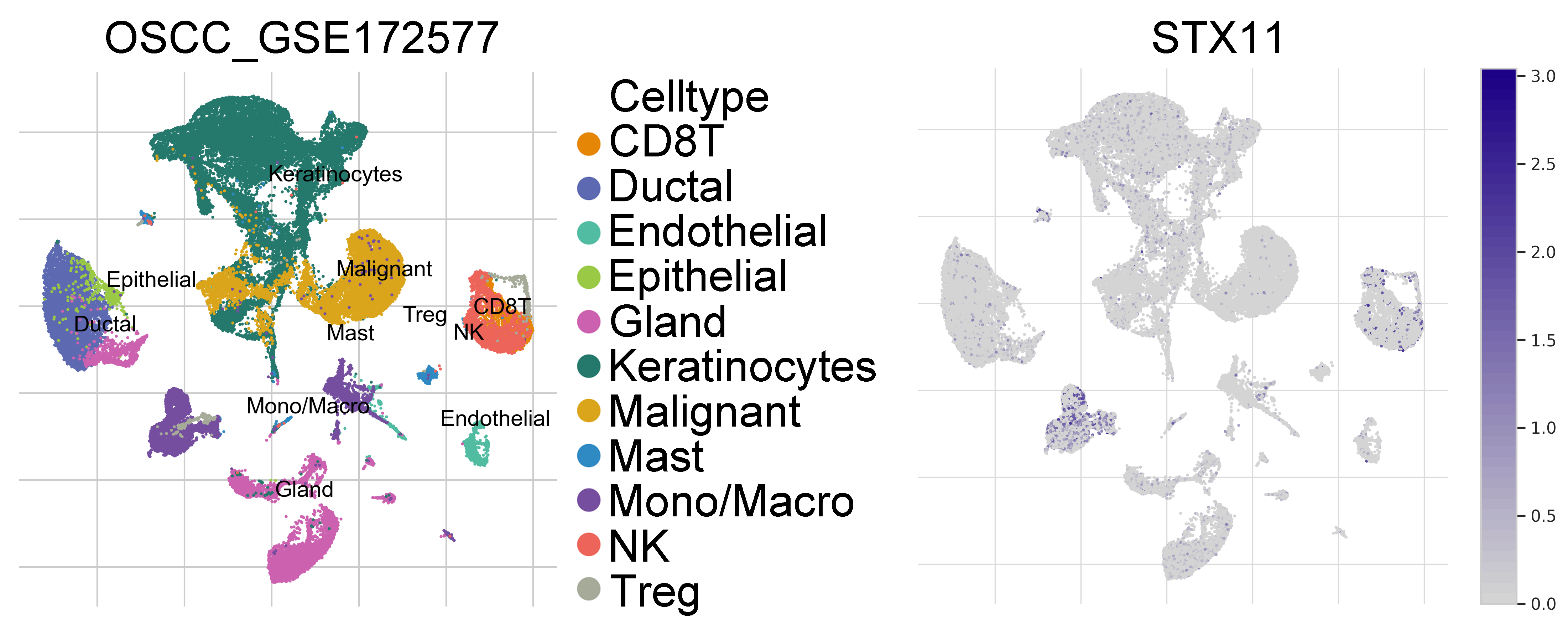
**Figure S15**. Single cell analysis of STX11 in oral squamous cell carcinoma.


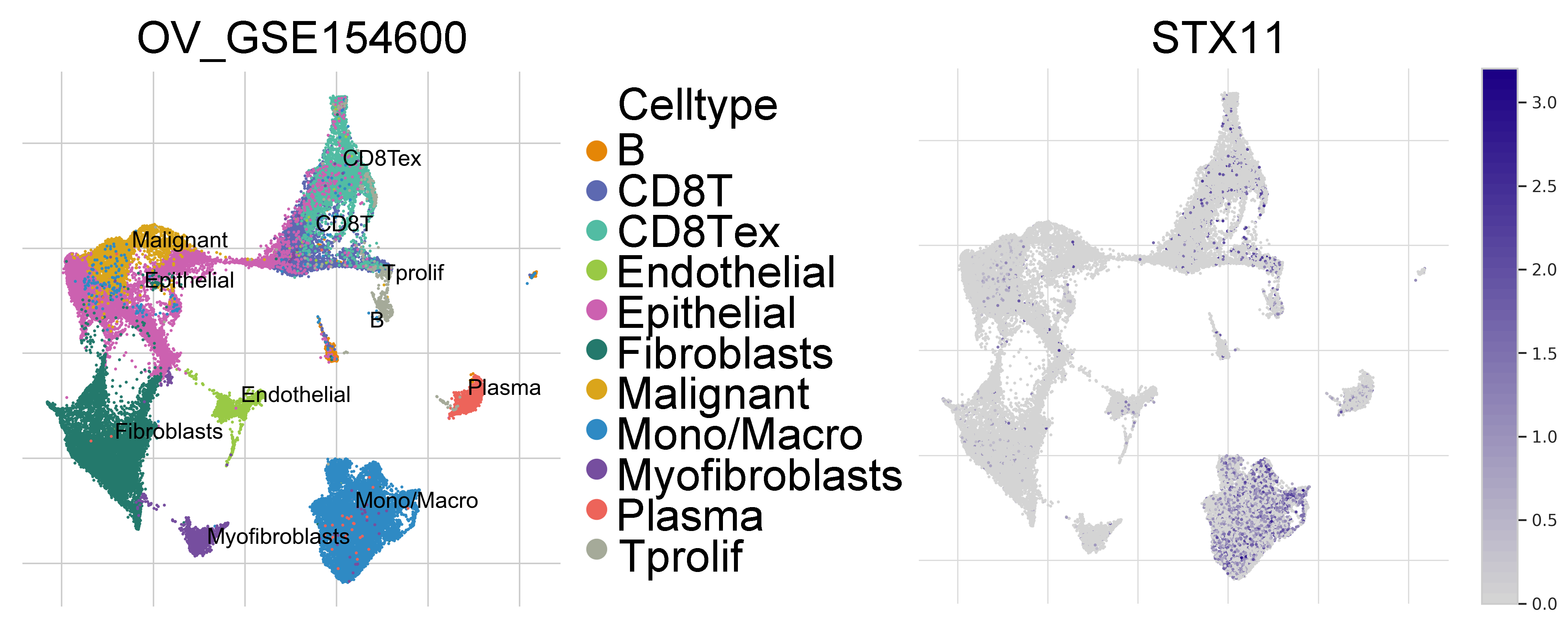
**Figure S16**. Single cell analysis of STX11 in ovarian cancer.


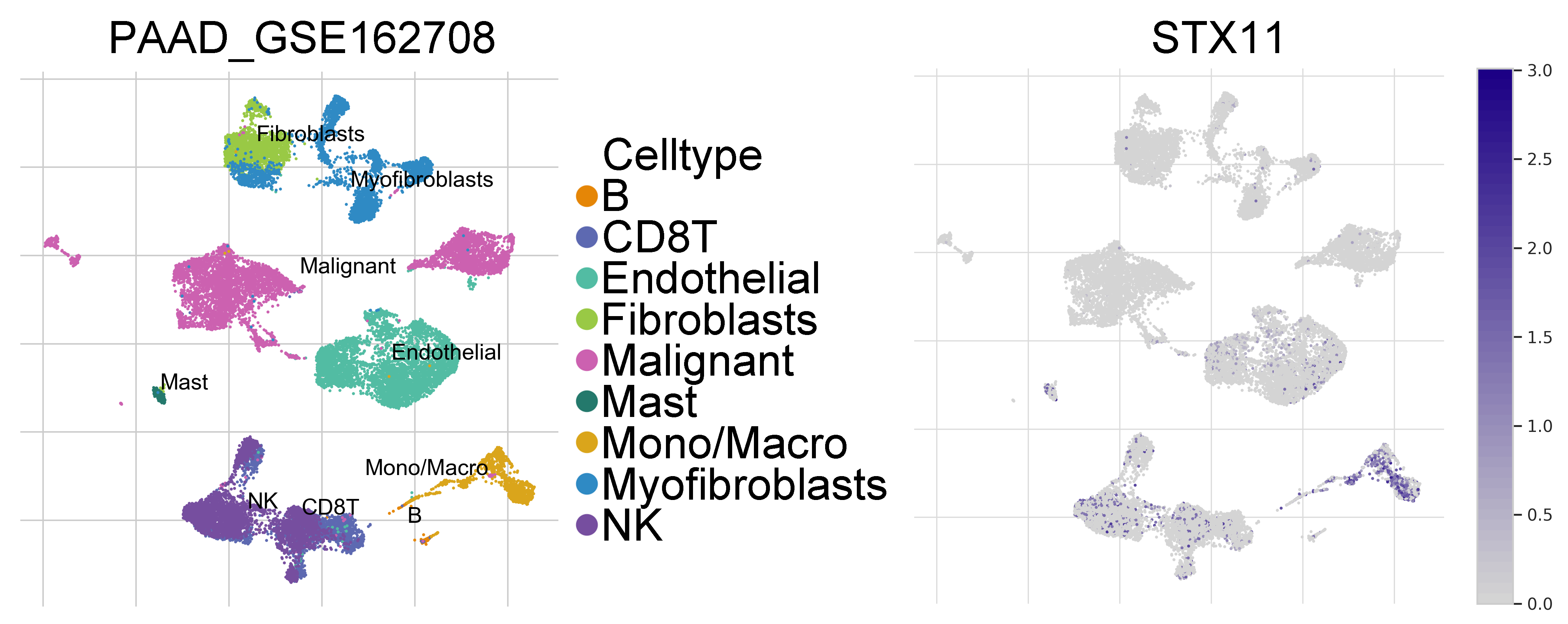


**Figure S17**. Single cell analysis of STX11 in pancreatic cancer.


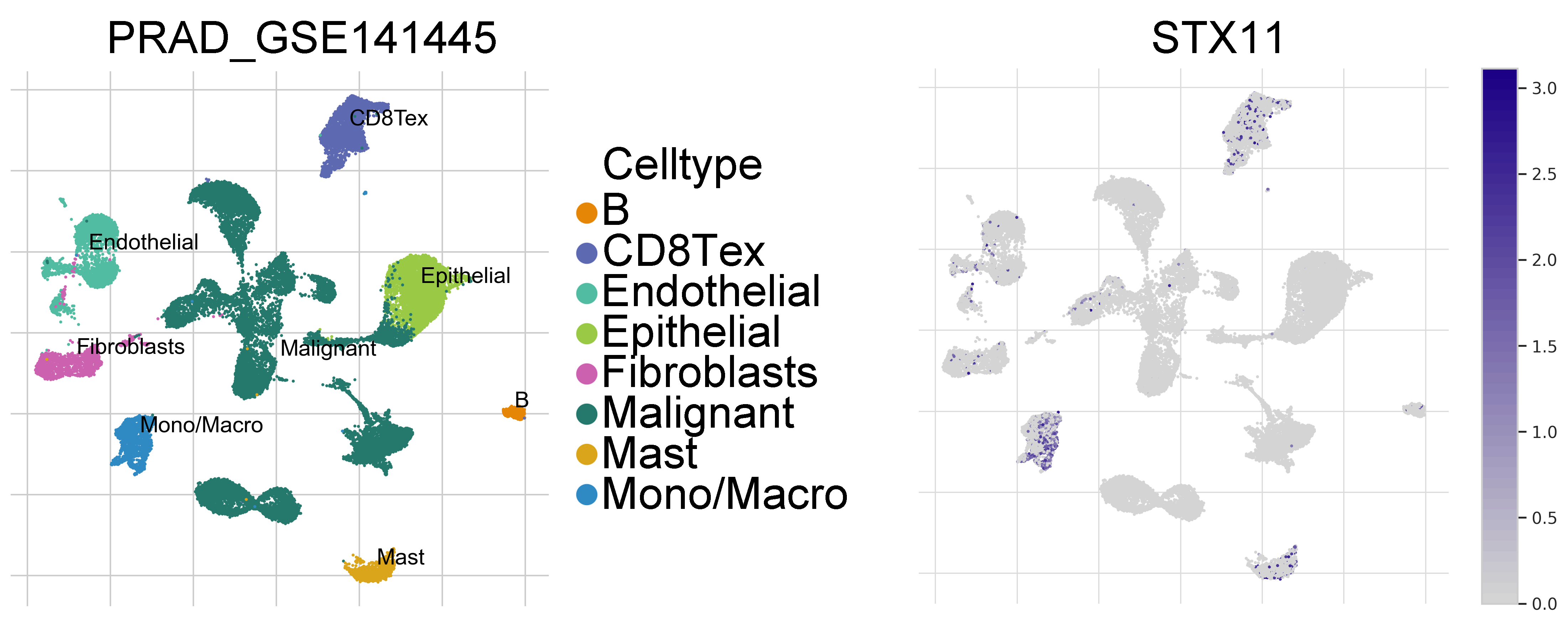


**Figure S18**. Single cell analysis of STX11 in prostate cancer.


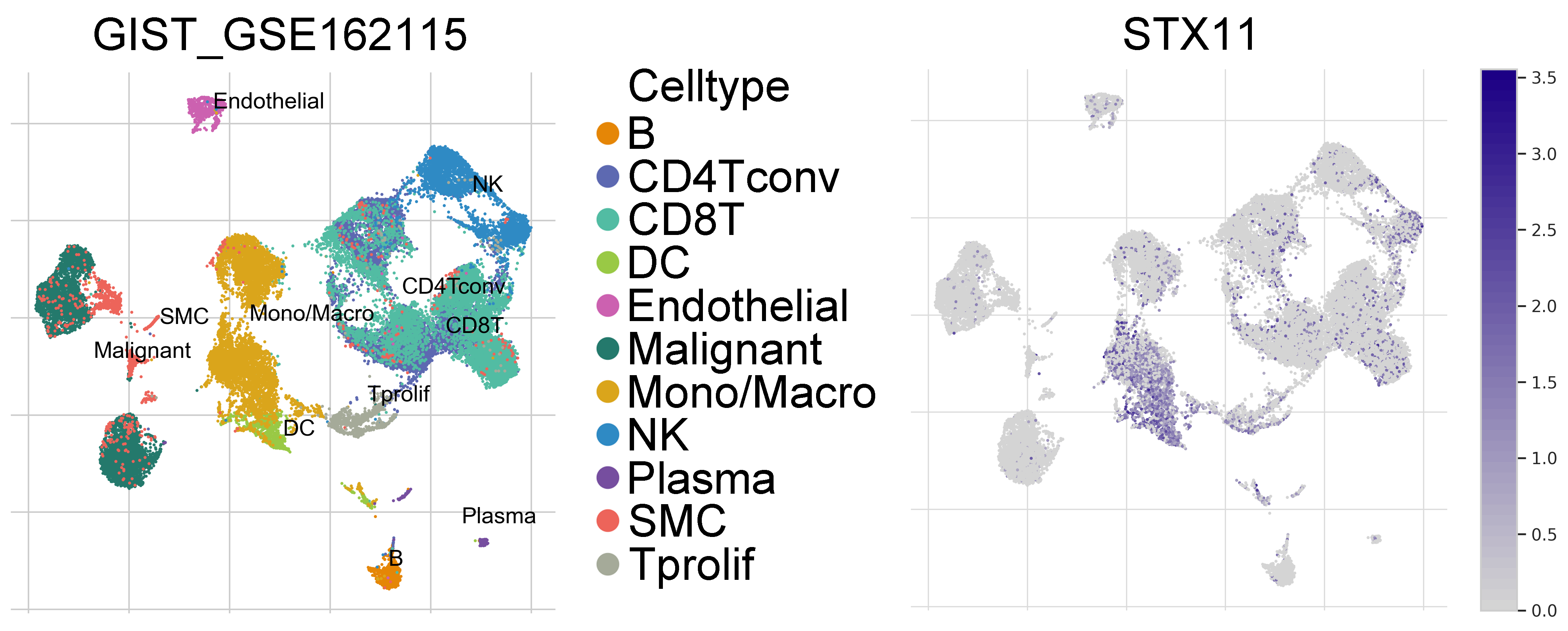
**Figure S19**. Single cell analysis of STX11 in gastrointestinal stromal tumor.

| Table S1. Primer for RT-qPCR | |
| --- | --- |
| STX11-F | CCCTCAACGAGATCGAGAGC |
| STX11-R | ACGTTGAGCTCGATGACGTT |
| IL1β-F | CTGTCCTGCGTGTTGAAAGA |
| IL1β-R | CTGCTTGAGAGGTGCTGATG |
| TNF-α-F | TCAACCTCCTCTCTGCCATC |
| TNF-α-R | CCAAAGTAGACCTGCCCAGA |
| IL10-F | TCTCCGAGATGCCTTCAGCAGA |
| IL10-R | TCAGACAAGGCTTGGCAACCCA |
| CCL22-F | ATGGATCGCCTACAGACTGC |
| CCL22-R | GGATCGGCACAGATCTCCTT |

| \| Table S2. Survival analysis of signatures in different breast cancer subtypes \| \| \| \| \| \| \| \| \| --- \| --- \| --- \| --- \| --- \| --- \| --- \| --- \| \|  \| Overall survival \| \| \|  \| Disease free survival \| \| \| \|  \| HR \| 95% CI \| P \|  \| HR \| 95% CI \| P \| \| Basal \| 2.131 \| 1.002-4.531 \| 0.049 \|  \| 1.389 \| 0.931-2.072 \| 0.107 \| \| HER2 \| 1.097 \| 0.523-2.299 \| 0.807 \|  \| 2.634 \| 1.516-4.577 \| 0.001 \| \| Luminal \| 2.963 \| 2.005-4.381 \| <0.001 \|  \| 2.034 \| 1.443-2.866 \| <0.001 \| \|  \|  \|  \|  \|  \|  \|  \|  \|   Table S3. Multivariate Cox analysis of overall survival | | | |
| --- | --- | --- | --- | --- | --- | --- | --- | --- | --- | --- | --- | --- | --- | --- | --- | --- | --- | --- | --- | --- | --- | --- | --- | --- | --- | --- | --- | --- | --- | --- | --- | --- | --- | --- | --- | --- | --- | --- | --- | --- | --- | --- | --- | --- | --- | --- | --- | --- | --- | --- | --- | --- | --- | --- | --- | --- | --- | --- | --- |
| Features | HR | 95% CI | P value |
| Age | 1.034 | 1.020-1.049 | <0.001 |
| T stage |  |  |  |
| T1 | Reference |  |  |
| T2 | 1.174 | 0.739-1.865 | 0.497 |
| T3 | 1.378 | 0.742-2.560 | 0.310 |
| T4 | 1.893 | 0.919-3.901 | 0.084 |
| N stage |  |  |  |
| N0 | Reference |  |  |
| N1 | 1.642 | 1.076-2.507 | 0.022 |
| N2 | 2.530 | 1.440-4.448 | 0.001 |
| N3 | 2.756 | 1.276-5.955 | 0.01 |
| M stage |  |  |  |
| M0 | Reference |  |  |
| M1 | 2.217 | 1.169-4.203 | 0.015 |
| riskScore | 2.072 | 1.528-2.809 | <0.001 |

Table footnote: For categorical variables, the reference group (HR = 1.0) is indicated. For continuous variables, HR represents per-unit change.

| Table S4. Multivariate Cox analysis of disease-free survival | | | |
| --- | --- | --- | --- |
| Features | HR | 95% CI | P value |
| T stage |  |  |  |
| T1 | Reference |  |  |
| T2 | 1.423 | 0.795-2.547 | 0.235 |
| T3 | 1.618 | 0.739-3.541 | 0.228 |
| T4 | 3.474 | 1.252-9.637 | 0.017 |
| N stage |  |  |  |
| N0 | Reference |  |  |
| N1 | 1.231 | 0.731-2.073 | 0.435 |
| N2 | 2.074 | 1.059-4.061 | 0.033 |
| N3 | 3.931 | 1.764-8.758 | 0.001 |
| M stage |  |  |  |
| M0 | Reference |  |  |
| M1 | 4.038 | 1.670-9.765 | 0.002 |
| PR |  |  |  |
| Negative | Reference |  |  |
| Positive | 0.532 | 0.276-1.026 | 0.060 |
| ER |  |  |  |
| Negative | Reference |  |  |
| Positive | 0.719 | 0.368-1.402 | 0.333 |
| riskScore | 1.570 | 1.148-2.146 | 0.005 |

Table footnote: For categorical variables, the reference group (HR = 1.0) is indicated. For continuous variables, HR represents per-unit change.
